# A concentrated array of copper porphyrin candidate qubits†
†Electronic supplementary information (ESI) available. CCDC 1871557. For ESI and crystallographic data in CIF or other electronic format see DOI: 10.1039/c8sc04435j


**DOI:** 10.1039/c8sc04435j

**Published:** 2018-11-21

**Authors:** Chung-Jui Yu, Matthew D. Krzyaniak, Majed S. Fataftah, Michael R. Wasielewski, Danna E. Freedman

**Affiliations:** a Department of Chemistry , Northwestern University , Evanston , Illinois 60208 , USA . Email: danna.freedman@northwestern.edu; b Institute for Sustainability and Energy at Northwester , Northwestern University , Evanston , Illinois 60208-3113 , USA

## Abstract

Synthetic chemistry offers a pathway to realize atomically precise arrays of qubits, the smallest unit of a quantum information science system. We harnessed framework chemistry to create an array of qubit candidates, featuring one qubit every 13.6 Å, by synthesizing the new copper(ii) variant of the porphyrinic metal–organic framework PCN-224. We subjected the framework to pulse-electron paramagnetic resonance (EPR) measurements, establishing spin coherence at temperatures up to 80 K within a fully spin concentrated framework. Observation of Rabi oscillations further support the viability of the qubits within these arrays. To interrogate the spin dynamics of qubit arrays, we investigated spin–lattice relaxation, *T*_1_, through a combination of pulse-EPR and alternating current (ac) magnetic susceptibility measurements. These data revealed distinct vibrational environments within the frameworks that contribute to spin dynamics. The aggregate results establish a pathway for a synthetic approach to create spatially precise networks of qubits.

## Abstract 2

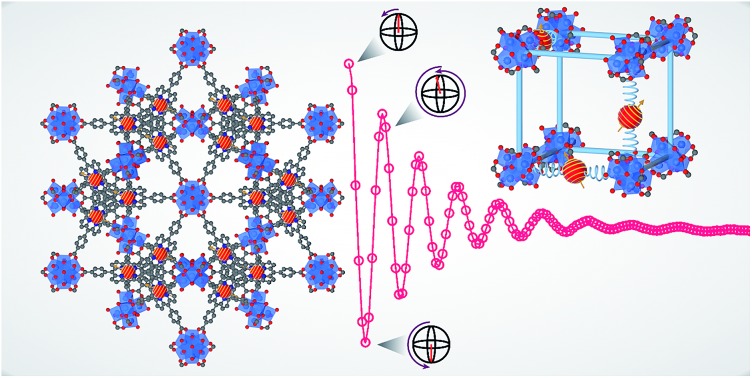
Metal–organic frameworks enable the synthesis of arrays of atomically precise qubits.

## Introduction

The advent of quantum information science (QIS) offers the potential to revolutionize our problem-solving approaches across fields, from cryptography to the simulation of protein folding.^1^–^5^ The development of qubits, the elementary units of a QIS system, is a prerequisite to realize the promise of QIS. Electronic spin sublevels (*M_S_* levels) in paramagnetic coordination complexes are a promising class of qubits due to the facile synthetic tunability of their chemical and magnetic parameters.^6^–^9^ In the development of this class of qubits, the performance can be determined by two metrics: the coherence time (*T*_2_), which is the superposition lifetime within which all computations must be performed,^9^ and the spin–lattice relaxation time (*T*_1_), which serves as the upper limit to *T*_2_ and the inverse of which (1/*T*_1_) determines the operating speed of the qubit.^10^ There is a rich body of work describing the spin dynamics of coordination complexes, which enabled the development of synthetic design principles for the creation of molecular qubits.^11^–^29^ These recent advances have propelled coordination complexes as viable qubit candidates for QIS, with spin properties comparable to those seen in solid-state materials, such as the heralded nitrogen vacancy pair defects in diamond.^14^,^29^,^30^

For coordination chemistry to realize QIS applications, the next crucial challenge is the integration of qubits into ordered arrays. Discrete spatial control is necessary to engender the qubit–qubit coupling for complex QIS system operations, such as gate operations within quantum computing.^7^,^9^,^31^,^32^ Much of the current studies on molecular qubits involve molecules dissolved in frozen solvent matrices or incorporated within diamagnetic solid-state matrices. The random distribution of spin centers within these matrices render discrete spatial control of qubits exceedingly difficult. Thus, there is a pressing need for the development of new materials that may incorporate molecular qubits into discrete ordered arrays.

Framework materials, most notably metal–organic frameworks (MOFs), offer a pathway to incorporate molecular qubits into well-ordered arrays. MOFs are porous materials synthesized from organic and inorganic building blocks using the fundamental principles of coordination chemistry.^33^–^36^ These materials hold several key advantages for the realization of ordered qubit arrays. First, the high degree of synthetic versatility of MOFs imparts immense synthetic control in the design of these materials, particularly for the installation of qubits. Furthermore, the highly modular nature of MOFs permits integration of two- and three-dimensional MOFs into an assortment of devices and substrates.^35^,^37^–^42^ Second, the porous nature of these materials spatially separates the qubits to potentially minimize destructive qubit–qubit interactions that shorten coherence time, thus permitting qubit manipulations in a concentrated array of qubits. In addition, the porosity of the framework offers the potential for quantum sensing applications. Quantum sensing would exploit the fragile superposition state of the qubit units to detect analytes within the framework.^43^

The aggregate of these features of MOFs suggest promise for the creation of qubit arrays in both QIS and quantum sensing applications. Indeed, a few recent studies have explored MOFs as platforms for such potential applications.^44^–^46^ Studies on copper(ii), vanadyl, and cobalt(ii) porphyrin frameworks have yielded new insights regarding the transition from molecular qubits to extended qubit arrays. However, these systems employed magnetically dilute MOFs, wherein only a fraction of the metal centers are spin active. The random nature of magnetic dilution results in similar limitations of spatial control of the spin centers to solution phase studies. The next step is to demonstrate spin coherence in a framework within which the spin centers are located at precise crystallographic positions. To achieve this, it is imperative to explore frameworks that are fully spin concentrated, thereby engendering spatial precision of the candidate qubits. Crucially, creating these networks enables quantification of the impact of spin–spin interactions on spin dynamics, which may provide new design principles for future molecular qubit arrays.

Herein we report the synthesis and pulse-EPR analysis of the new materials Cu_0.1_-PCN-224 (**1**), Cu_0.4_-PCN-224 (**2**), and Cu_1.0_-PCN-224 (**3**), which are new variants of the known Zr-based porphyrinic MOF PCN-224 featuring *S* = ½ copper(ii) centers bound by the porphyrin linkers. Copper(ii) porphyrins have been studied extensively within the EPR literature,^47^–^52^ and their coherence properties are well established in molecular form. The ability to easily compare the spin dynamics of copper(ii) porphyrins embedded in MOFs to molecular copper(ii) porphyrins is important for extracting the contribution of the MOF lattice to spin dynamics. We demonstrate the successful incorporation of the copper(ii) porphyrin within the PCN-224 lattice. Crucially, we observe spin coherence up to 80 K in the fully spin concentrated framework **3**. To better understand the contributions of electron–electron spin interactions and the phonon environment of the MOF on spin–lattice relaxation time, *T*_1_, and on coherence times, *T*_2_, we subjected these materials to a series of measurements. Pulse-EPR spectroscopy and ac magnetic susceptibility measurements enabled us to quantify the different contributions of electron–electron spin interactions and the phonon environment of the MOF on *T*_1_, thereby suggesting future design principles for networks of electronic spin candidate qubits.

## Results and discussion

**Fig. 1:**
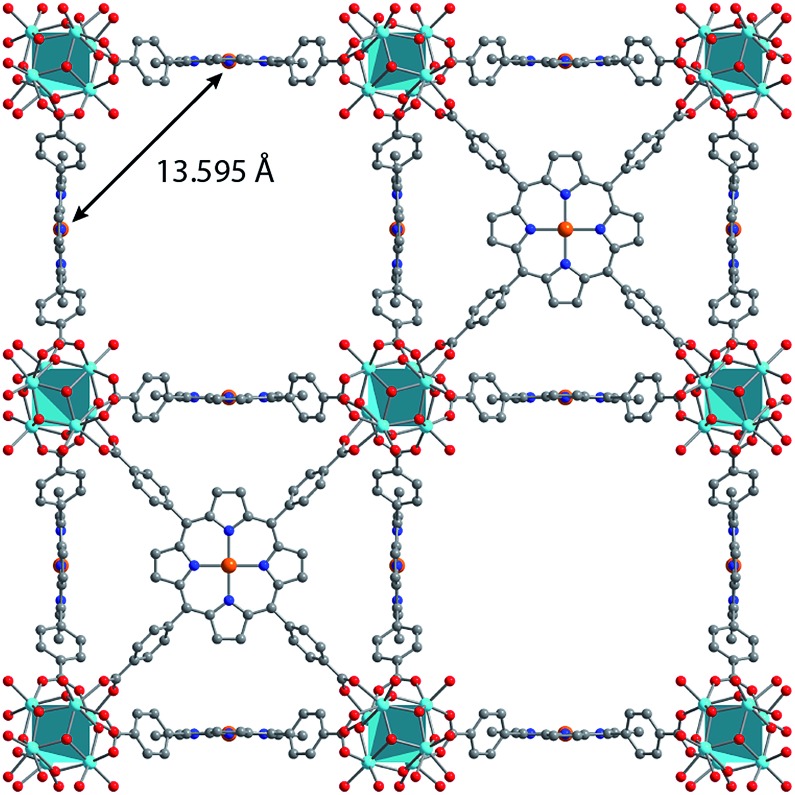
Crystal structure of the new material Cu_1.0_-PCN-224 (**3**) with each porphyrinic ligand metalated with Cu^2+^. Orange, red, blue, and gray represent copper, oxygen, nitrogen, and carbon, respectively. [Zr_6_O_6_] nodes are represented by the turquoise polyhedra. Nearest neighbor distances between Cu^2+^ centers are highlighted.

Synthesis of MOFs **1–3** proceeded *via* hydrothermal reactions of a mixture of zirconium tetrachloride (ZrCl_4_), 5,10,15,20-tetrakis(carboxyphenyl)porphyrin copper(ii) (CuTCPP), 5,10,15,20-tetrakis(carboxyphenyl)porphyrin (TCPP), and benzoic acid in dimethylformamide (DMF). CuTCPP and TCPP were mixed in the desired molar ratio to achieve the targeted degree of metalation. Incorporation of CuTCPP into the framework was confirmed by diffuse-reflectance UV/visible spectroscopy and inductively coupled plasma-optical emission spectroscopy (ICP-OES). N_2_ adsorption isotherms of **1–3** yielded BET surface areas of 2427–3076 m^2^ g^–1^, confirming the porosity of the material. (ESI, Fig. S16–S19†). Single crystal X-ray diffraction of **3** confirmed the PCN-224 structure with copper(ii) porphyrinic ligands, with nearest Cu–Cu distances of 13.5948(2) Å (Fig. 1). Continuous-wave (CW) EPR spectroscopy on **1–3** was modelled using EasySpin^53^ with axial *g* and *A* components, yielding *g*_‖_ = 2.186, *g*_⊥_ = 2.042, *A*Cu‖ = 611 MHz, *A*Cu⊥ = 79.4 MHz, *A*N‖ = 43.2 MHz and *A*N⊥ = 48.2 MHz (Fig. S1, Table S2†). We observe a concentration dependence with the Lorentzian linewidth *Γ*_L_ of the CW spectra, with line broadening increasing with copper(ii) concentration as a result of electron–electron dipolar interactions.^54^ The *g* and *A* values are consistent with those observed in molecular copper porphyrins.^47^,^50^,^52^ Thus, we confirm the successful incorporation of molecular copper(ii) porphyrins into the PCN-224 framework with little change in electronic structure, thereby permitting our study of molecular spin dynamics within a MOF matrix.

**Fig. 2:**
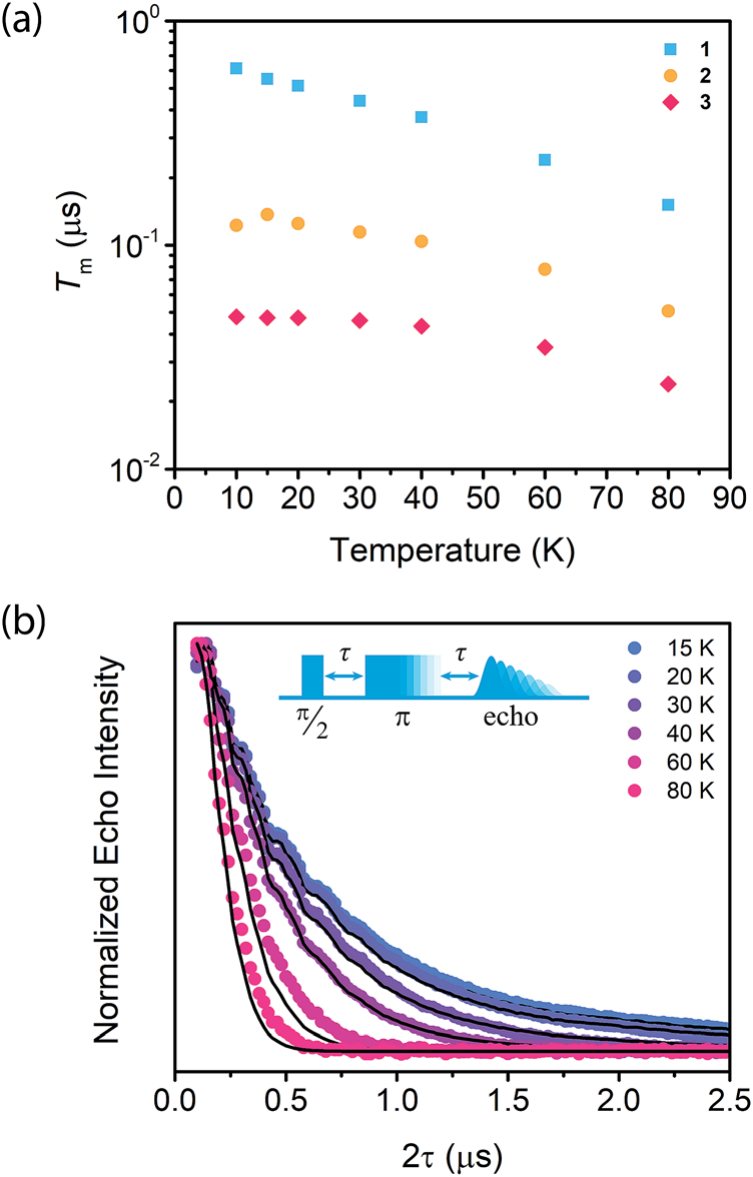
(a) Temperature dependence of *T*_m_ of 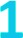, 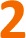, and 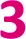, collected at 3328 G for 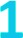 and 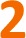 and at 3410 G for 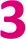 from 10–80 K. The Hahn Echo pulse sequence used to measure *T*_m_ is shown at the bottom. (b) Representative fits to the Hahn echo decay of **1** at 2942 G across 15–80 K based on our model incorporating electron–electron spin distances and *T*_1_.

To begin our investigation on the spin dynamics of **1–3**, we first sought to measure the phase memory time (*T*_m_) of the copper(ii) centers in the PCN-224 framework. *T*_m_ encompasses all processes that contribute to electron spin decoherence, including the intrinsic coherence time *T*_2_ of the electron spin.^55^ Here, we were particularly interested in whether we could observe a spin echo with **3**, the fully concentrated framework, due to the spin dense environment. To quantify *T*_m_, Hahn echo experiments were performed on the resonances at 2942 G and 3328 G across 10–80 K for **1–3**. The two resonances correspond to different molecular orientations as a result of the axial environment of the copper(ii) spin center. The lower field resonance corresponding to the principle axis *g*_‖_, which is perpendicular to the porphyrin plane. The higher field resonance corresponds to a powder average of orientations. Fits to a monoexponential decay collected on the 3328 G resonance yielded *T*_m_ values of 645, 121, and 46 ns for **1–3**, respectively, at 10 K. By 80 K, *T*_m_ decays to 158, 38, and 25 ns for **1–3**, respectively (Fig. 2a). Similar values were seen for **1–3** at 2942 G (Fig. S3†). Remarkably, in **3** a spin echo was observed up to 80 K at both resonances.

The foregoing results prompted us to test the quantum mechanical behavior of **1–3** through the application of a transient nutation.^56^–^59^ Rabi oscillations were observed for all of the frameworks by measuring the projection of the magnetization onto the *z* axis with a Hahn echo as a function of the duration of an initial nutation pulse. As expected, the nutation frequencies scale with the applied microwave powers in accordance with the *S* = ½ spin state for **1–3** (see Fig. S12†).^56^,^60^ The observation of Rabi oscillations and the *S* = ½ nature of the spins in **1–3** establish them as viable arrays of candidate qubits.

Compounds **1–3** demonstrate a clear trend in *T*_m_ between the different spin concentrations (Fig. 2a, S3†) across 10–80 K, whereby *T*_m_ sequentially decreases in magnitude with increasing spin concentration. It is noteworthy that a nearly identical trend in the temperature dependence on *T*_m_ is observed across **1–3**. At both resonances, *T*_m_ does not depend significantly on temperature up to 20 K, after which *T*_m_ decreases at approximately the same rate between **1–3** (Fig. 2a, S3†). Within **1–3**, the crystalline structure and open channels of PCN-224 yield identical nuclear spin environments around the copper(ii) spin centers. The contribution of the fluctuating hyperfine field from nuclear spins to *T*_m_ should be the same between **1–3**.^54^ Given that the only difference between **1–3** is the concentration of spins, we expect an enhancement in *T*_m_ relaxation induced by electron dipole–dipole interaction with increasing spin concentration. This process occurs when random copper(ii) spin flips, the rate of which is inversely proportional to *T*_1_, promote *T*_m_ relaxation to nearby, dipolar-coupled copper(ii) spin centers.^61^ We sought to model the temperature dependence of *T*_m_ for **1–3** incorporating electron–electron dipolar relaxation process. To do so, we applied the theory developed by Salikhov *et al.* that describes the contribution of electron dipole–dipole interactions and *T*_1_ to the electron spin echo decay (see ESI†).^61^

For our model, we utilized both the copper(ii)–copper(ii) distances obtained from the crystal structure of **3** up to 60 Å and a weighted number of spins (Table S14†). Utilizing the distances and the measured *T*_1_'s, *vide infra*, we calculated the *T*_m_ relaxation enhancement relative to 10 K as a function of temperature (see ESI† for further details). Our model yielded fits that reproduce the temperature dependence of *T*_m_ (Fig. 2b, S6†), and we find that approximately 50 Å represents the limit at which the contribution of this relaxation process is significant. This distance is well within the range of electron–electron spin distances explored with electron–electron double resonance (ELDOR) techniques, suggesting the 50 Å limit from this model is within reason.^62^–^65^ Previous works on nitroxide radicals have demonstrated that, for distances beyond 60 Å, nuclear spin-induced decoherence pathways become much more significant than electron–electron spin interactions, further supporting the 50 Å limit determined for **1–3** by our model.^63^,^65^ Taken together, the 50 Å benchmark proposed by this model may serve as a key design parameter for future developments of ordered qubit arrays in MOFs. Additionally, this model provides a method to quantify electron–electron spin distances and interactions even in a highly spin concentrated system with *T*_1_ and *T*_m_ measurements, as ELDOR techniques are limited by the short *T*_m_ lifetimes.

We then proceeded to quantify *T*_1_ in **1–3** across 10–80 K. Fundamentally, spin–lattice relaxation is the phonon-mediated equilibration of the excited and ground Zeeman energy levels for an electronic spin. In addition to phonon processes, spin–lattice relaxation may also be induced by spin–spin interactions through cross relaxation, whereby energy is exchanged from the observed copper(ii) spin center to another, fast-relaxing center mediated by dipolar interactions.^66^ This relaxation pathway will possess a characteristic relaxation rate that contributes to the saturation recovery experiment, in addition to the intrinsic spin–lattice relaxation rate. Thus, the magnitude of the cross relaxation rate, relative to that of spin–lattice relaxation, will provide insight to the competition between *T*_1_ relaxation induced by environmental phonons or spin–spin interactions.

**Fig. 3:**
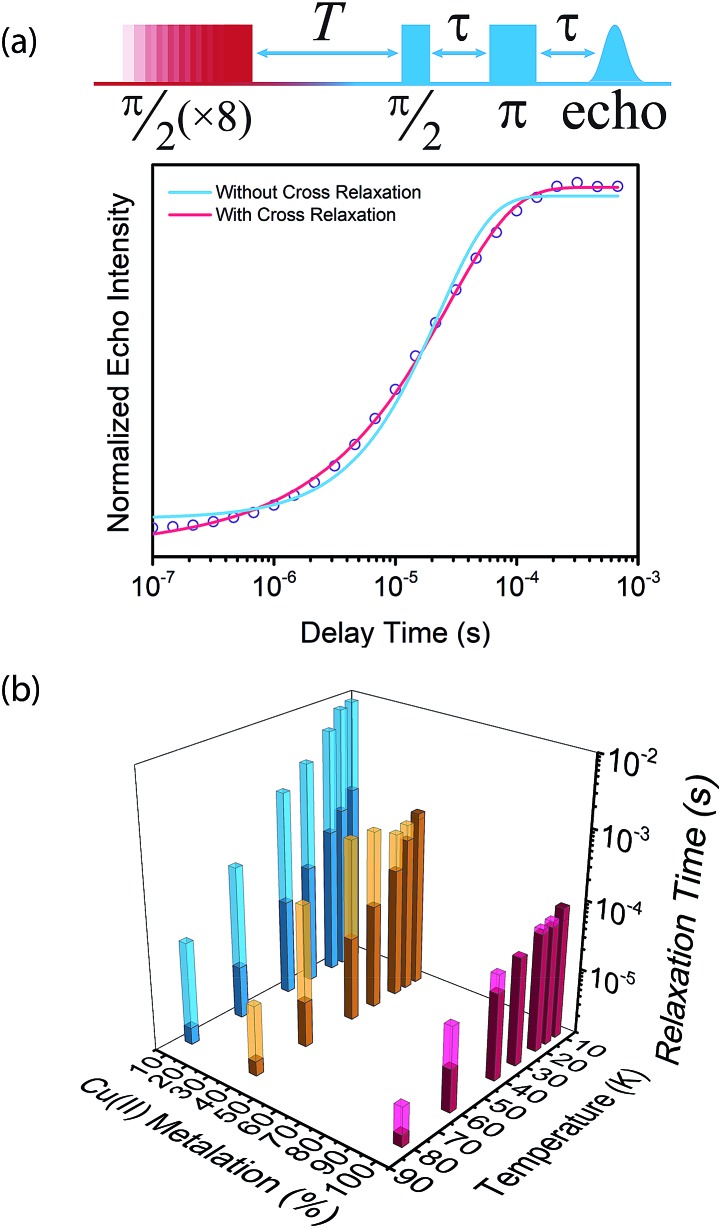
(a) Normalized saturation recovery curve of 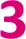 measured on the 3410 G resonance at 20 K, including the schematic of the pulse sequence. The magenta and blue lines are fits to the data with and without incorporating cross relaxation, respectively. (b) *a* and *T*_1_ values of 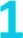, 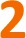, and 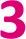 on the 3328/3410 G resonance. Lighter bars represent relaxation times by cross relaxation (*a*), whereas the darker bars represent *T*_1_ relaxation times.

**Fig. 4:**
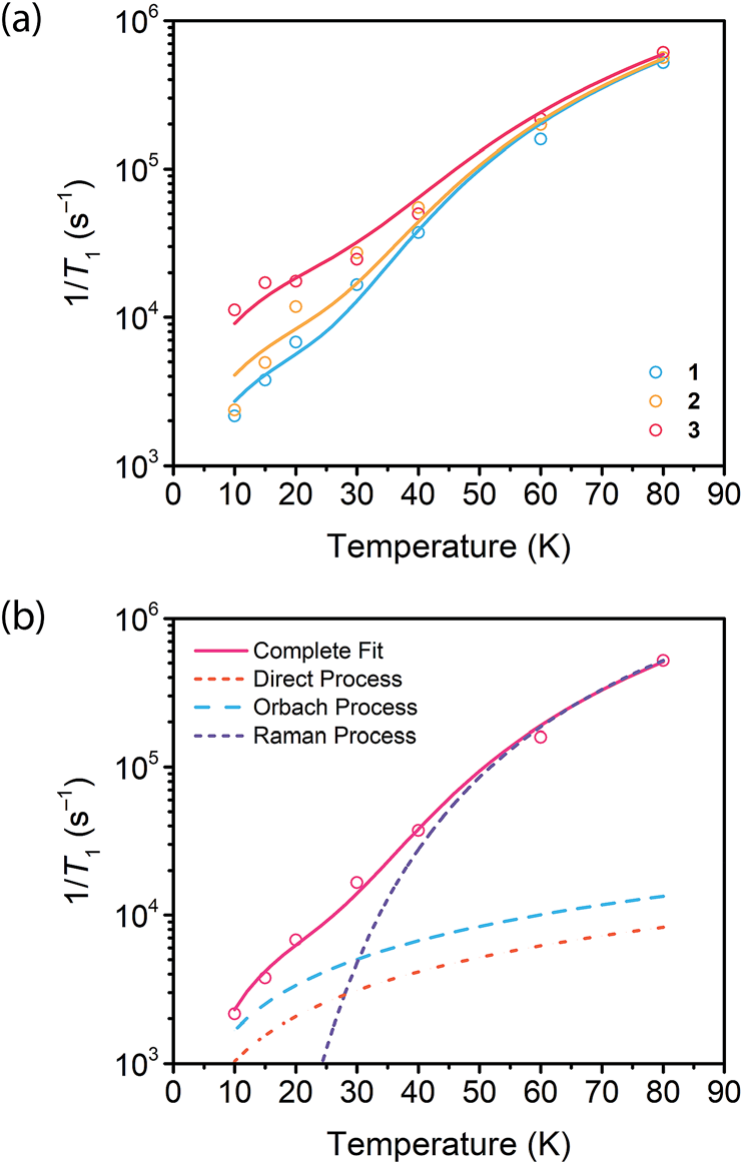
(a) Fits to the temperature dependence of 1/*T*_1_ for 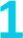, 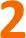, and 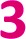 at 3328 G. Results of the fits are reported in Table S13.† (b) Fit to the temperature dependence of 1/*T*_1_ of **1** at 3328 G resonance. The red line represents the total fit, while the orange, blue and purple dashed lines represent the contributions of the direct, Orbach–Aminov and Raman processes, respectively.

Saturation recovery experiments on **1–3** at 2942 and 3328 G yielded *T*_1_ valuesshown in Fig. 4a and S4.† The saturation recovery experiments on **1–3** were fit with the following expression (Fig. 3a, see ESI†):
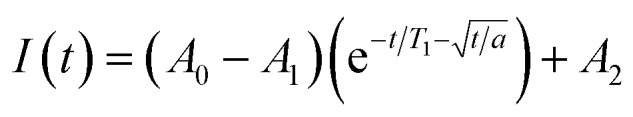
which, in addition to *T*_1_, included the time associated with relaxation induced by cross relaxation (*a*). With this expression, the extracted *T*_1_ values are the intrinsic spin–lattice relaxation times of the samples, with the contribution of cross-relaxation separately accounted for in *a*. In the idealized case, a pulse train is designed to saturate the resonance line providing a monoexponential relaxation rate *T*_1_. In **1–3**, the saturation recovery curves exhibit non-exponential behavior that is adequately modeled with cross relaxation using the equation above (Fig. 3a). The ratio *a*/*T*_1_ was calculated for **1–3** to quantitate cross relaxation relative to *T*_1_ (Fig. 3b, S9, S10†). A clear concentration dependence on cross relaxation is observed across **1–3**, with *a*/*T*_1_ varying two orders of magnitude between **1** and **3**. The large ratios of *a*/*T*_1_ in **1** across 10–80 K reveal cross relaxation to be a minor relaxation mechanism relative to phonon-mediated processes. In contrast, *a*/*T*_1_ on the order of 1 in **3** across the entire temperature range suggests cross relaxation becomes a significant mechanism of relaxation and must be considered alongside other, phonon-mediated relaxation processes. Taken together, the concentration dependence of spectral diffusion highlights spin–spin interactions as a critical parameter in dictating *T*_1_.

We then assessed the temperature dependence of *T*_1_ across **1–3** to explore phonon contributions to *T*_1_ relaxation. Within 10–80 K, we observe a distinct concentration dependence in the spin–lattice relaxation rate (1/*T*_1_) across **1–3** at 3328 G, while no dependence was observed at the 2942 G resonance (Fig. 4a, S11†). To investigate this concentration dependence, we employed a model that accounts for three main phonon processes that contribute to *T*_1_ relaxation, which are the direct, Raman and Orbach–Aminov processes (Fig. 4b). The direct process is a low energy, single phonon process that is typically dominant at temperatures below 10 K. The Raman process is a two-phonon event analogous to the Raman scattering of photons, and is often dominant in the temperature range of 20–100 K in analogous molecular porphyrin complexes.^67^ Similarly, the Orbach–Aminov process is also a two-phonon process, with the difference that the process is facilitated by a specific excited state of the system. This latter process may manifest from spin concentrated systems, wherein dipolar interactions between electronic spins generate low-lying excited states that facilitate energy transfer for spin–lattice relaxation.^66^,^68^

To quantify the contribution of each process in Cu-PCN-224, we globally fit temperature dependence of *T*_1_ for **1–3** at both resonances (Fig. 4a, S11, ESI†). For the Orbach process, the energy of the excited state was calculated based on the through-space dipole–dipole interaction between nearest neighbor copper(ii) spin centers (13.595 Å). From the results of the fits, the values of the contributions of the direct (*A*_dir_) and Raman (*A*_Ram_) processes are approximately one and two orders of magnitude larger than those obtained for copper tetratolylporphyrin (CuTTP) diluted in a diamagnetic ZnTTP matrix (Tables S12, S13†).^49^ Given the similarity in *g* factors between **1–3** and CuTTP in a ZnTTP matrix, we can rule out differences in spin–orbit coupling, which couples lattice phonons to the electronic spin, as the source of this difference in phonon relaxation pathways. To explain the discrepancy, we propose the MOF lattice contributes a different vibrational environment that may facilitate spin relaxation more effectively. Indeed, a recent work has demonstrated new vibrational environments of vanadyl porphyrins emerging in a MOF lattice, in contrast to a diamagnetic solid state molecular matrix.^45^ In **1–3**, the MOF lattice likely possess unique low-energy phonons that enhance *T*_1_ relaxation rates relative to their molecular analogues, thus accounting for the enhanced influence of the direct and Raman processes in **1–3**. The results of the fits also revealed a concentration dependence in the values of the Orbach process (*A*_Orb_) across **1–3** at 3328 G. This concentration dependence has been similarly observed in previous studies on trityl radicals and Ir^4+^ ions in single crystals.^66^,^68^ The observation of this concentration dependence suggests that dipolar-coupled copper(ii) spin centers may act as a fast-relaxing center that facilitate *T*_1_ relaxation of nearby spin centers. Curiously, no similar dependence was observed at the 2942 G resonance. This may suggest a potential orientation dependence on the contribution of the Orbach relaxation process on *T*_1_. At 2942 G, the EPR transition probed along the *g*_‖_ orientation may experience weaker dipolar interactions with neighboring spin centers. The EPR transition at 3328 G, which encompasses a powder average of orientations, may experience the more dipolar interactions with nearby spin centers, including those at 13.595 Å away.

**Fig. 5:**
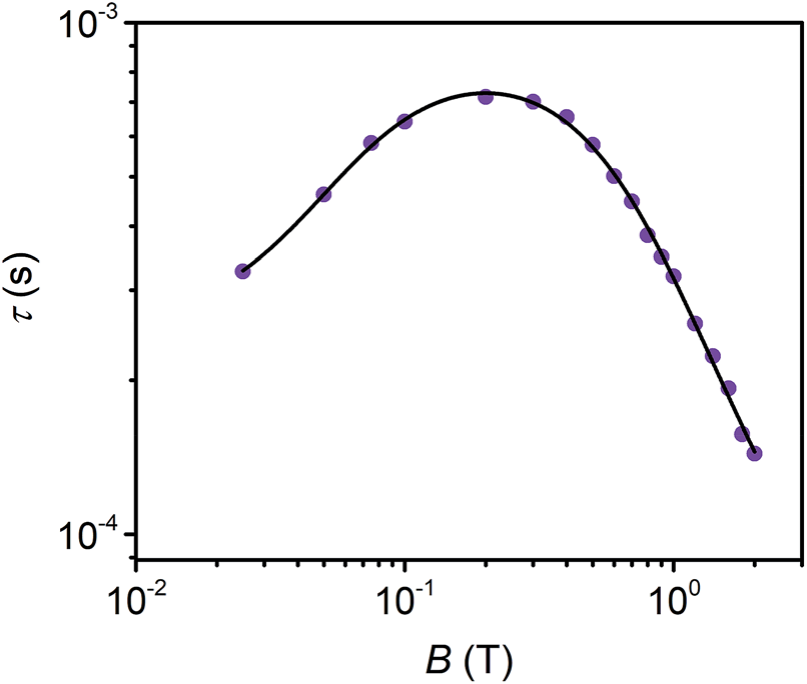
Field dependence of *τ* extracted from ac susceptibility measurements of **3** at 5 K. The black line represents fits to the data with the extended Brons–van Vleck model.

Field dependent ac susceptibility of **3** provides an additional probe to the interplay between spin–spin cross relaxation and phonon mediated relaxation processes. Real (*χ*′) and imaginary (*χ*′′) components of the ac signal were measured across 0.025–2 T at 5 K, and the relaxation time, *τ*, was extracted by fitting Cole–Cole plots of the data (see ESI†). The field dependence of *τ* reveals an increase in *τ* from 0.025–0.2 T, with the maximum at 0.72 ms, before decaying from 0.2–2 T. The origin of this behavior stems from the suppression of spin–spin and spin–nuclei interactions with increasing magnetic fields, tempered by the increase in the efficiency of the direct process at higher fields.^12^,^13^,^30^,^69^ To quantify the contribution of the direct process to *τ*, we fit the field dependence of *τ* in **3** with the extended Brons–van Vleck model:^12^,^13^ 
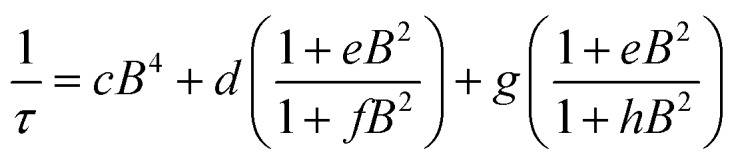
where the first term relates to the field dependence of the direct process. The second and third terms account for the effect of the internal magnetic field that contributes to spin relaxation, including inter- and intramolecular interactions such as spin–nuclei hyperfine and spin–spin dipolar interactions. The results of the fit are reported in Fig. 5 and Table S18.† The large value of *c* in **3** suggests a large contribution by the direct process in facilitating spin relaxation, corroborating our findings from pulse-EPR that the MOF lattice may possess more phonon modes with energies that greatly decrease *T*_1_.^12^,^13^,^70^ This is additionally supported by the occurrence of the maximum *τ* at lower fields relative to previously investigated molecular species, suggesting a greater density of low energy phonons that mediate spin relaxation *via* the direct process.^12^,^13^,^49^ These results direct us to consider utilizing magnetic fields to suppress cross relaxation to lengthen *T*_1_, and consequently *T*_2_, and reaffirm the distinct phonon environment of the Cu-PCN-224 lattice.

## Outlook

The foregoing results demonstrate the utility of synthetic chemistry to create spatially precise, concentrated arrays of qubits. We synthesized a new variant of the framework PCN-224 and studied the spin dynamics of the *S* = ½ centers within. Vitally, we observed spin coherence in a fully spin concentrated network, furthering the promise of MOFs as an architecture for incorporating molecular qubits in ordered arrays. We employed a model for *T*_2_ that, taking into account electron–electron spin interactions and *T*_1_, proposed a 50 Å limit within which spin–spin interactions play a significant role in decoherence. Through pulse-EPR and ac susceptibility measurements, distinct phonon environments in MOFs from their molecular analogues are observed. The sum of this work emphasizes *T*_1_ as a key figure of merit in limiting decoherence in spin–dense arrays. Future studies exploring different organic linkers and MOF structures on phonon distributions and electron–electron coupling strength in MOFs will be imperative to propel the field of molecular qubits towards scalable architectures.

## Conflicts of interest

There are no conflicts of interest.

## Supplementary Material

Supplementary information

Crystal structure data
