# Supplementary material for: A concentrated array of copper porphyrin candidate qubits
Source: Chem Sci. 2018 Nov 21;10(6):1702–8. doi: 10.1039/c8sc04435j (PMC6368214; doi:10.1039/c8sc04435j)
Supplement: Supplementary file 1 [file SC-010-C8SC04435J-s001.pdf]

Electronic Supplementary Material (ESI) for Chemical Science.
This journal is © The Royal Society of Chemistry 2018

Supporting Information for:

A Concentrated Array of Copper Porphyrin Candidate Qubits

Chung-Jui Yu,[†] Matthew D. Krzyaniak,[†,§] Majed S. Fataftah,[†] Michael R. Wasielewski,*[†,§] and Danna E. Freedman*[,†]

[†]*Department of Chemistry, Northwestern University, Evanston, Illinois 60208, United States*

[§]*Argonne-Northwestern Solar Energy Research (ANSER) Center, Northwestern University, Evanston, Illinois, 60208–3113, United States*

Table of Contents

**Full Experimental Details**		**S4**
**Table S1 |**	Full crystallographic table for **3**	**S12**
**Table S2 |**	Spin Hamiltonian parameters for **1–3**.	**S13**
**Table S3–S5 |**	T_2 relaxation times and fit parameters for **1–3**.	**S14**
**Table S6–S7 |**	T_1 relaxation times and fit parameters for **1**.	**S15**
**Table S8–S9 |**	T_1 relaxation times and fit parameters for **2**.	**S16**
**Table S10–S11 |**	T_1 relaxation times and fit parameters for **3**.	**S17**
**Table S12 |**	Fit parameters for the $1/T_1$ temperature dependence of **1–3** at 2942/3010 G.	**S18**
**Table S13 |**	Fit parameters for the $1/T_1$ temperature dependence of **1–3** at 3328/3410 G.	**S18**
**Table S14 |**	Fit parameters for the Hahn echo decay of **1–3** based on spin-spin interaction model.	**S18**
**Table S15–S16 |**	Fit parameters for variable temperature ac susceptibility of **3** at 2940 G and 3330 G.	**S19**
**Table S17 |**	Fit parameters for variable field ac susceptibility of **3** at 5 K.	**S19**
**Table S18 |**	Fit parameters for the field dependence of τ of **3**.	**S20**
**Table S19 |**	Tabulated fit parameters to calculate BET surface areas of **1–3**.	**S20**
**Table S20 |**	Variable-power nutation frequencies of **1–3** collected at the 2942/3010 resonance at 20 K.	**S20**
**Figure S1 |**	CW EPR spectra for **1–3**.	**S21**
**Figure S2 |**	Echo-detected EPR spectra for **1–3**.	**S22**
**Figure S3 |**	Variable temperature T_2 data for **1–3** at 2942/3010 G.	**S23**
**Figure S4 |**	Variable temperature T_1 data for **1**–**3** at 2942/3010 G and 3328/3410 G.	**S24**
**Figure S5 |**	Echo decay curves of **1–3** at 2942/3010 G and 3328/3410 G.	**S25**
**Figure S6 |**	Echo decay curves of **1**–**3** at 2942/3010 G and 3328/3410 G with fits from spin-spin interaction model.	**S26**
**Figure S7 |**	Saturation recovery curves of **1–3** with spectral diffusion fits at 2942/3010 G and 3328/3410 G.	**S27**
**Figure S8 |**	Saturation recovery curves of **1–3** with stretched fits at 2942/3010 G and 3328/3410 G.	**S28**
**Figure S9 |**	Temperature dependence of a and T_1 of **1–3** at 2942/3010 G.	**S29**
**Figure S10 |**	Temperature dependence of a/T_1 at 2942/3010 G and 3328/3410 G.	**S30**
**Figure S11 |**	Fits to the temperature dependence of T_1 of **1–3** at 2942/3010 G.	**S31**

**Figure S12 |**	Variable-B_1 nutation data of **1**–**3**, Fourier transforms of the oscillations, and B_1-dependence of nutation frequency at 2942/3010 G at 20 K.	**S32**
**Figure S13 |**	Temperature dependence of τ for **3**.	**S33**
**Figure S14 |**	Variable temperature Cole-Cole plots and fits of **3** at 2940 G and 3330 G.	**S34**
**Figure S15 |**	Variable field Cole-Cole plots and fits of **3** at 5 K.	**S35**
**Figure S16 |**	Diffuse-reflectance UV-vis spectra for **1–3**.	**S36**
**Figure S17 |**	N_2 adsorption isotherms of **1–3**.	**S37**
**Figure S18 |**	BET consistency plots of **1–3**.	**S38**
**Figure S19 |**	Plots of $1/[v(P_0/P{-}1)]$ vs P/P_0 and fits for **1–3**.	**S39**
**References**		**S40**

Experimental Details

General Considerations. Unless otherwise noted, all solvents and reagents were purchased from commercial vendors and used without further purification. Zirconium tetrachloride ($ZrCl_4$) was purchased from Sigma-Aldrich and stored under an inert atmosphere. All MOF syntheses were executed in Qorpak™ clear borosilicate sample vials GLC01006. MOF syntheses were performed on the benchtop, and after washing the synthesized MOFs were stored under an inert atmosphere. The material [5,10,15,20-Tetrakis(4-carboxyphenyl)porphyrinato]-Cu(II) (CuTCPP) was synthesized according to literature procedures.[1] The preparation of H_2TCPP was performed following the previously described procedure.[2,3]

Note on the synthesis of X% Cu-PCN-224. We found that the purity of $ZrCl_4$ was crucial to synthesizing highly crystalline MOF. Exposure of $ZrCl_4$ to air and solvents caused the white powder to turn yellow over time, which can lead to MOFs of poor crystallinity. In addition, water was found to be crucial to selectively forming the PCN-224 structure over other porphyrin MOF structure types. Commercial DMF does not have the required water content to selectively form PCN-224, and hence 1–3 drops of DI water were added to each MOF synthesis vial. We were able to successfully synthesize phase-pure materials reported below with the amount of water stated.

10% Cu-PCN-224 (1). We combined $ZrCl_4$ (2.52 g, 10.8 mmol) and 168 mL of DMF in a 500 mL Erlenmeyer flask and then sonicated the mixture for an hour to yield a clear colorless solution. Benzoic acid (72 g, 590 mmol) was then added, and the mixture was sonicated for an hour to form a clear, light yellow solution. We placed CuTCPP (5.3 mg) and H_2TCPP (65.1 mg) into each of the 12 GLC01006 vials and evenly distributed the Zr solution among the 12 vials (~19 mL each, 49.1 mmol benzoic acid, 0.90 mmol $ZrCl_4$). We then added one drop of water to each vial. The vials were sealed and sonicated for an hour to yield dark green solutions. The vials were heated in an oven at 130 °C for three days, after which dark precipitates formed along the vial walls. After cooling to room temperature, the dark red supernatant was decanted from each vial. A sample of the material from each vial was washed with 2 × 2 mL acetone, and then screened by PXRD. All vials with phase-pure PCN-224 were combined in a 200 mL jar, and the MOF was washed with 6 × 150 mL DMF at 150 °C and 6 × 150 mL methanol at 80 °C. The MOF was then dried under vacuum at 150 °C overnight, and subsequently transferred into a N_2 glovebox. The MOF was activated in accordance to the procedure below, then stored in a solvent-free glovebox. Experimental BET surface area of 3030 m^2/g closely matches those reported of other metallated PCN-224, confirming the successful activation and porosity of the framework.[4] ICP-OES confirmed the degree of CuTCPP incorporation into the framework. Yield: 0.1292 g (3.1%). Expected Zr:Cu ratio from ICP-OES: 40:1; Found: 43.8:1.

40% Cu-PCN-224 (2). We combined $ZrCl_4$ (2.52 g, 10.8 mmol) and 168 mL of DMF in a 500 mL Erlenmeyer flask and sonicated the mixture for an hour to yield a clear colorless solution. Benzoic acid (72 g, 590 mmol) was then added, and the mixture was sonicated for an hour to form a clear, light yellow solution. We added CuTCPP (27.2 mg) and H_2TCPP (44.8 mg) to each of the 12 GLC01006 vials and distributed the Zr solution evenly among the 12 vials (~19 mL each, 49.1 mmol benzoic acid, 0.90 mmol $ZrCl_4$). One drop of water was added to each vial. The vials were sealed and sonicated for an hour to yield dark green solutions. The vials were heated in an oven at 130 °C for three days, after which dark precipitates formed along the vial walls. After cooling to room temperature, the dark red supernatant was decanted from each vial. A sample of the material from each vial was washed with 2 × 2 mL acetone, and then screened by PXRD. All vials with phase-pure PCN-224 were combined in a 200 mL jar, and the MOF was washed with 6 × 150 mL DMF at 150 °C and 6 × 150 mL methanol at 80 °C. The MOF was then dried under vacuum at 150 °C overnight, and subsequently transferred into a N_2 glovebox. The MOF was activated in accordance to the procedure below, then stored in a solvent-free glovebox. Experimental BET surface area of 2427 m^2/g closely matches those reported of other metallated PCN-224, confirming the successful activation and porosity of the framework.[4] ICP-OES confirmed the degree of CuTCPP incorporation into the framework. Yield: 0.4852 g (11.4%). Expected Zr:Cu ratio from ICP-OES: 10:1; Found: 9.35:1.

100% Cu-PCN-224 (3). We combined $ZrCl_4$ (2.52 g, 10.8 mmol) and 168 mL of DMF in a 500 mL Erlenmeyer flask and sonicated the mixture for an hour to yield a clear colorless solution. Benzoic acid (72 g, 590 mmol) was then added, and the mixture was sonicated for an hour to form a clear, light yellow solution. We added CuTCPP (56.6 mg) and H_2TCPP (17.5 mg) to each of the 12 GLC01006 vials and distributed the Zr solution evenly distributed among the 12 vials (~19 mL each, 49.1 mmol benzoic acid, 0.90 mmol $ZrCl_4$). One drop of water was added to each vial. The vials were sealed and sonicated for an hour to yield dark green solutions. The vials were heated in an oven at 130 °C for three days, after which dark precipitates formed along the vial walls. Single crystals of **3** were also present within the precipitates yielded from this hydrothermal synthesis and were washed and activated with the bulk of the yielded material. After cooling to room temperature, the dark red supernatant was decanted from each vial. A sample of the material from each vial was washed with 2 × 2 mL acetone, and then screened by PXRD. All vials with phase-pure PCN-224 were combined in a 200 mL jar, and the MOF was washed with 6 × 150 mL DMF at 150 °C and 6 × 150 mL methanol at 80 °C. The MOF was then dried under vacuum at 150 °C overnight, and subsequently transferred into a N_2 glovebox. The MOF was activated in accordance to the procedure below, then stored in a solvent-free glovebox. Experimental BET surface area of 3076 m^2/g closely matches those reported of other metallated PCN-224, confirming the successful activation and

porosity of the framework.[4] ICP-OES confirmed the degree of CuTCPP incorporation into the framework. Yield: 0.4752 g (10.9%). Expected Zr:Cu ratio from ICP-OES: 4:1; Found: 3.99:1.

Electron Paramagnetic Resonance Measurements

We transferred samples of the activated MOFs into 4 mm OD Wilmad quartz tubes in a solvent-free nitrogen glovebox. To prevent dioxygen contamination, we attached the tubes onto a custom-built adapter for the Micromeritics ASAP 2020 instrument in the glovebox. The setup was then transferred from the glovebox to the instrument, enabling an air-free transfer. The EPR tube was cycled four times with ultra-high purity helium, then approximately 450 mmHg He was dosed into the tube. We then flame-sealed the tube. For 100% Cu-PCN-224, the MOF was transferred into a 1.6 mm OD Wilmad Q-band quartz tube, which was then placed inside the 4 mm quartz tube and sealed.

All pulse and cw EPR data for the MOFs were collected at X-band frequency (9–10 GHz) on a Bruker Elexsys E580 EPR spectrometer (Bruker Biospin, Rheinstetten, Germany) equipped with a split ring resonator (ER4118X-MS5) and employed a 1 kW TWT amplifier (Applied Systems Engineering) at Northwestern University. Temperature was controlled via an Oxford Instruments CF935 helium cryostat and an Oxford Instruments MercuryiTC temperature controller. Continuous wave (cw) EPR spectra were collected at 80 K for **1**–**3**. The spectra were fit using Easyspin.[5] The spectra were fit using a spin system containing an $S = \frac{1}{2}$ electronic spin coupled to a Cu nucleus, assuming natural isotopic abundance of ^{63}Cu ($I = \frac{3}{2}$) and ^{65}Cu ($I = \frac{3}{2}$), and four ^{14}N nuclei ($I = 1$) to generate the multiline patterns seen in the cw EPR spectra. Linewidths were fit with Gaussian and Lorentzian distributions, the latter of which was used to determine line broadening due to electron-electron dipolar interactions.[6] Parameters for each fit to the cw spectra are reported in Table S2. Based on the symmetry of the Cu site in the porphyrin unit, we constrained the hyperfine coupling values for the four nitrogen nuclei to be identical. The parameters for **1**–**3** are consistent with those of other molecular porphyrin complexes, indicative that no significant change in electronic structure incurred upon incorporation into a MOF.[7–10]

For pulse EPR measurements, we selected two resonances at which we performed our pulsed EPR experiments: 2942 G and 3328 G. For **3**, the same resonances appeared at 3010 G and 3410 G, respectively. The EPR transitions at these two field positions represent transitions along two different molecular orientations. The lower field transition corresponds to the principal axis $g_{\parallel}$, which is perpendicular to the porphyrin plane. The higher field transition corresponds to a powder average of orientations.[10–12] Previous studies have established orientation dependences of spin relaxation in copper(II) porphyrins, including a recent work on the spin dilute 2D copper(II) porphyrinic MOF $[\{Cu_{0.01}Zn_{0.99}TCPP\}Zn_2(H_2O)_2]_\infty \cdot 2DMF$.[13–15] T_1 and T_2 between the two resonances in **1**–**3** were very similar, in accordance to the orientation dependence explored in a previous study on a copper(II) porphyrinic MOF.[15]

Pulsed measurements were conducted at the two resonances for **1–3**. In performing the pulsed measurements, pulse lengths and delays were adjusted to maximize echo intensity. Typical pulse sequences were used as follows; Echo decay curves to determine T_2 were collected by application of a two-pulse echo sequence ($\pi/2 - \tau - \pi - \tau -$ echo) with increasing interpulse delay τ. Pulse lengths of the $\pi/2$ and π pulses were 16 and 32 ns, respectively, with an initial interpulse delay of 100 ns. All T_2 measurements used 4-step phase cycling. Saturation recovery curves to determine T_1 were collected by application of a picket-fence sequence of eight microwave pulses of 20 ns with an interpulse delay of 1 μs. This was followed with a two-pulse echo sequence ($\pi/2 - \tau - \pi - \tau -$ echo) with 16 and 32 ns pulse lengths for the $\pi/2$ and π pulses, respectively, and an initial interpulse delay of 100 ns. Field-swept echo-detected EPR spectra were collected using a two-pulse echo sequence with microwave pulses of 16 and 32 ns with a 100 ns delay time between the pulses. Transient nutation experiments were performed at 20 K for **1–3** at the 2942 G resonance with a three-pulse sequence ($t_p - T - \pi/2 - \tau - \pi - \tau -$ echo). T and τ were held constant at 600 and 100 ns, respectively. The duration of the $\pi/2$ and π pulses were 16 and 32 ns, respectively, for all microwave powers.

Echo decay curves and nutation data were phased by maximization of the sum of the real component of the signal and minimization of the sum of the imaginary component of the signal. Saturation recovery curves were phased by the maximization of the sum of the squares of the real component of the signal and minimization of the sum of the squares of the imaginary component of the signal. Data manipulations were performed in Origin 9.0 and MATLAB R2016a. To extract spin-lattice relaxation times (T_1), saturation recovery curves were fit using a function that accounts for the contribution of cross relaxation (a): $I(t) = (A_0 - A_1)\left(e^{-t/T_1 - \sqrt{t/a}}\right) + A_2$.[16] As a comparison, the data was also fit with the stretched exponential function: $I(t) = \left(1 - A_0 e^{-(t/T_1)^{\beta}}\right) + A_1$. The value of the stretch factor β correlates to the range of relaxation rates observed in the saturation recovery curve. For two dominant relaxation processes that contribute to T_1 relaxation, the value of β should approach 0.5. We observe β values of 0.66 at 10 K in **3**, which increases slightly to 0.83 at 80 K. In contrast, the β values of **1** remain from 0.87–0.92 across the entire temperature range. This suggests that spectral diffusion relaxation process is significant in **3**, corroborating the findings found through the fit incorporating spectral diffusion. Spin-spin relaxation times (T_2) were determined by fitting echo decay curves to a monoexponential function: $I(2\tau) = A_0 e^{-2\tau/T_2} + A_1$. Nutation data were normalized and zero-filled with 1024 data points prior to performing a fast Fourier transformation with a Hanning window.

To model the contribution of spin-spin interactions in T_2, the modulations of the Hahn echo decays of **1–3** was fit using a custom MATLAB function based on the following function:[17,18]

$$V(2\tau) = \left\{\left(\cos(R\tau) + \left(\frac{1}{2T_1 R}\right)\sin(R\tau)\right)^2 + \left(\frac{\omega_{d-d}}{2R}\right)^2 \sin^2(R\tau)\left(1 - 2\left\langle \sin^2\left(\frac{\varphi}{2}\right)\right\rangle\right)\right\} e^{-\tau/T_1}$$

$$R = \frac{\omega_{d-d}}{2}\sqrt{1 - \frac{1}{(\omega_{d-d}T_1)^2}}$$

$$\omega_{d-d} = \frac{1}{\hbar}\left(\frac{g_1 g_2 \beta^2 (1 - 3cos^2\theta)}{r^3}\right)$$

where T_1 is the spin-lattice relaxation time, $\omega_{d\text{–}d}$ is the frequency of the dipolar interaction between two spin centers, $\langle \sin^2\left(\frac{\varphi}{2}\right)\rangle$ is the probability of the excitation of nearby dipolar-coupled spins by the applied microwave pulse, g_1 and g_2 are the g-factors of the dipolar-coupled spins, r is the distance between the dipolar coupled spins, and θ is the angle between the direction of the applied magnetic field and that of r. To apply this model for the temperature dependence of T_2 in **1–3**, we first determined the copper(II)-copper(II) distances within the framework using the crystal structure of **3**, which are reported in Table S13. With these distances and the T_1 values of **1–3**, we fit the Hahn echo decay curves with the following function:

$$I(T, 2\tau) = I(10\mathrm{K}, 2\tau)\left(\frac{V(T, r_1, 2\tau)}{V(10K, r_1, 2\tau)}\right)^{m_1(C)^{f_1}}\left(\frac{V(T, r_2, 2\tau)}{V(10K, r_2, 2\tau)}\right)^{m_2(C)^{f_2}} \cdots \left(\frac{V(T, r_n, 2\tau)}{V(10K, r_n, 2\tau)}\right)^{m_n(C)^{f_n}}$$

Fundamentally, we sought to isolate the temperature-dependent contributions to spin-spin interactions by taking the ratio between the calculated modulations $V(T, r_i, 2\tau)$ for a given spin-spin distance r_i at some temperature T and 10 K, which serves as the reference spectrum. Doing so determines the Hahn echo decay modulation by spin-spin interactions at distance r_i for a given temperature T relative to that at 10 K. We calculate this ratio for an n number of copper(II)-copper(II) distances r_1–r_n. The exponent $m_i(C)^{f_i}$ accounts for the number of copper(II)-copper(II) distances (m_i), the concentration of copper(II) centers in the framework (C), and a weight factor f_i that accounts for the magnitude of the contribution by spin centers present at the distance r_i for spin concentrations less than 100%. As f_i approaches 0, $(C)^{f_i}$ will approach unity, suggesting that the spins at that distance are contributing significantly to spin relaxation. In contrast, as f_i becomes large, $(C)^{f_i}$ will approach 0, suggesting spins at that distance contribute little to spin relaxation. After calculating the temperature-dependent contributions at each distance r_i, each contribution was combined with the Hahn echo decay of the reference 10 K data, thereby including temperature independent contributions to spin decoherence such as electron spin echo envelope modulation (ESEEM) and intrinsic T_2 of the spin centers. We thus apply this equation to model the Hahn echo decay of the system at the temperature T. In applying this model, the Hahn echo decays of **3** at both resonances were fit simultaneously, varying m_i to determine the number of contributions that are present at a given r_i. The upper limit for these values were set to be the number of copper(II)-copper(II) distances determined from the crystal structure. These m_i values were then applied to fit the Hahn echo decays of **1** and **2**. All data sets of **1** and **2** were fit globally, varying the weight factor f_i to fit the data. Copper(II)-copper(II) distances up to

60 Å were considered in our model. Previous works involving spin labels have demonstrated dipolar interactions between electronic spins up to 10 nm.[19–21] For our model, distances beyond 60 Å did not significantly improve the fit of the experimental data, suggesting that the contribution of these distant spins to T_2 relaxation are not significant. Results of the fit are reported in Table S13 and Figure S7.

The temperature dependence of T_1 was fit using the curve fitting application in MATLAB R2016a with the following equation:[22]

$$\frac{1}{T_1} = A_{Dir}T + A_{Ram}\left(\frac{T}{\theta_D}\right)^9 J_8\left(\frac{\theta_D}{T}\right) + A_{Orb}\left[\frac{1}{e^{\theta_{Orb}/T-1}}\right]$$

where T is the temperature in Kelvin, A_{dir} is the contribution from the direct process, A_{Ram} is the contribution from the Raman process, θ_{D} is the Debye temperature, A_{Orb} is the contribution from the Orbach-Aminov process, θ_{Orb} is the Orbach energy corresponding to the energy of the excited energy state, and J_8 is the transport integral:

$$J_8\left(\frac{\theta_D}{T}\right) = \int_0^{\theta_D/T} x^8 \frac{e^x}{(e^x-1)^2}\,dx$$

The full analytical expression of the transport integral used in fitting (and written in the form of MATLAB code) is:

$J_8(x)$ = real(-(*x*.^8./(-1+exp(*x*)))+8.*(-(*x*.^8./8)+*x*.^7.*log(1-exp(*x*))+7.*x.^6.*polylog(2,exp(*x*))-42.*x.^5.*polylog(3,exp(*x*))+210.*x.^4.*polylog(4,exp(*x*))-840.*x.^3.*polylog(5,exp(*x*))+2520.* x.^2.*polylog(6,exp(*x*))-5040.*x.*polylog(7,exp(*x*))+5040.*polylog(8,exp(*x*)))-8.* (5040.*polylog(8,1)))

where $x = \left(\frac{\theta_D}{T}\right)$. θ_{Orb} was calculated using the point-dipole approximation and assuming $g = 2$. Nearest neighbor spin-spin distance of 13.5948 Å was used for the calculation. All T_1 vs. temperature data for a given resonance were fit simultaneously across **1–3**, with only the A_{Orb} parameter varying between the fits across **1–3**.

Magnetometry. All samples were prepared under an inert atmosphere in a glove box and were measured in sealed polyethylene pouches. Samples of **3** were densely packed into polyethylene pouches, heat sealed, and rolled to fit into plastic straws to prevent torqueing of crystallites. All measurements were conducted on a Quantum Design MPMS-XL SQUID magnetometer. Initially, magnetization from 0–3 T at 80 K was measured, and linear fit to the data indicated no ferromagnetic impurities in the sample. Ac susceptibility measurements were performed with a 4 Oe ac field oscillating at frequencies between 1–1488 Hz under various applied dc fields (0.025–2 T) and temperatures (5–10 K). The obtained data was corrected for diamagnetic contributions from the polyethylene pouch as well as core diamagnetism from the sample using

Pascal's constants.[23] Cole-Cole plots of the ac susceptibility data were fit with the following equations to extract τ:[24]

$$\chi'(\omega) = \chi_S + (\chi_T - \chi_S)\frac{1 + (\omega\tau)^{1-\alpha}\sin\left(\frac{\pi\alpha}{2}\right)}{1 + 2(\omega\tau)^{1-\alpha}\sin\left(\frac{\pi\alpha}{2}\right) + (\omega\tau)^{2-2\alpha}}$$

$$\chi''(\omega) = (\chi_T - \chi_S)\frac{(\omega\tau)^{1-\alpha}\cos\left(\frac{\pi\alpha}{2}\right)}{1 + 2(\omega\tau)^{1-\alpha}\sin\left(\frac{\pi\alpha}{2}\right) + (\omega\tau)^{2-2\alpha}}$$

In the Cole-Cole plots of the ac susceptibility data collected at 3410 G, a shoulder appears at high frequency (500–1488 Hz), which can be attributed to a second relaxation process. We fitted the dominant relaxation process to extract out τ values.

The magnetic field dependence of τ was fit with the extended Brons-van Vleck model shown in the following equation:[25–27]

$$\frac{1}{\tau} = cB^4 + d\frac{1 + eB^2}{1 + fB^2} + g\frac{1 + eB^2}{1 + hB^2}$$

The first term of the equation relates to the field dependence of the direct process. The second and third terms account for the effect of the internal magnetic field that contributes to spin relaxation, which includes inter- and intramolecular interactions such as spin-spin dipolar interactions.

τ values obtained from ac susceptibility as a function of temperature (Figure S13b) reveal spin relaxation times comparable to those obtained from pulse EPR, with τ = 446 μs and T_1 = 89 μs at 10 K. Discrepancies may be attributed to spin-phonon bottleneck effects that slow spin relaxation due to poor dissipation of phonons generated by spin relaxation.[28,29]

Activation and Surface Area Measurements. Samples of **1–3** were packed into a pre-weighed analysis tube and sealed with a Micromeritics TranSeal™. Activation and measurement were performed on a Micromeritics ASAP 2420 instrument. The samples were activated at 150 °C, with an initial ramp rate of 3 °C, until an outgas rate of ≤ 1 μmHg/min was observed. The sample tube was then weighed to determine the final mass of the MOF. Nitrogen adsorption isotherms were collected at 77 K. Surface areas were calculated from the nitrogen adsorption isotherms using Brunauer-Emmett-Teller (BET) theory. Optimal P/P_0 pressure ranges used to calculate BET surface area was determined through a plot of $v(P_0–P)$ vs P/P_0 in accordance to the first BET consistency criterion, where v is the volume of N_2 adsorbed per gram of MOF.[30] P/P_0 values at and before the maximum of $v(P_0–P)$ were used to calculate the BET surface area, as shown in Figure S18. After these measurements, the samples were immediately transferred to a solvent-free nitrogen glovebox for storage.

X-ray Data Collection, Structure Solution and Refinement for 3. The crystal structure of **3** was collected at the X-ray Crystallography lab of the Integrated Molecular Structure Education and Research Center at Northwestern University. Crystals of **3** were coated in Paratone N oil and mounted on MiTeGen MicroMounts™ rods under a stream of N_2 at 100 K. Crystallographic data for **3** were collected at 100 K on a Bruker KAPPA diffractometer with a CuKα IμS microfocus X-ray source with MX Optics, Apex II detector, and Oxford Cryosystems Cryostream cryostat. All raw data were integrated and corrected for Lorentz and polarization effects using Bruker Apex2 v. 2014.11.[31] Absorption corrections were applied using SADABS.[32] The space group of **3** was determined by examination of systematic absences, *E*-statistics, and successive refinement of the structure. The crystal structures were solved with direct methods and further refined with SHELXL[33] operated with the OLEX2 interface.[34] Thermal parameters for all non-hydrogen atoms were refined anisotropically. All hydrogen atoms were fixed at ideal positions, refined using a riding model for all structures, and refined using isotropic displacement parameters derived from their parent atoms. Full crystallographic details of **3** are listed in Table S1. Residual electron density found in the difference Fourier map was removed using the solvent mask protocol in OLEX2. This residual electron density likely arises from either residual solvent, or partial occupation of a separate morphology of a previously reported and closely related compound MOF-525.[35]

Other Physical Measurements. UV-Visible diffuse reflectance spectra were recorded on an Agilent Cary 5000 UV/Vis/NIR spectrometer equipped with an integrating sphere. Samples were prepared by a 7-fold dilution of the MOF with $BaSO_4$ and pulverized with an agate mortar and pestle to yield a smooth, homogenous powder. The spectra were treated with a background correction of $BaSO_4$. ICP-OES measurements were collected with a Thermo iCAP 7600 instrument. Elemental analysis was performed at the Integrated Molecular Structure Education and Research Center (IMSERC) at Northwestern University. The determined elemental percentages are as follows: Anal. Calcd. for $C_{144}Cu_{0.3}H_{121.4}N_{12}O_{64}Zr_{12}$ (**1**): 41.60 %C, 2.94 %H, 4.04 %N. Found: 46.74 %C, 2.72 %H, 3.96 %N. Anal. Calcd. for $C_{144}Cu_{1.2}H_{119.6}N_{12}O_{64}Zr_{12}$ (**2**): 41.05 %C, 2.86 %H, 3.99 %N. Found: 48.15 %C, 3.02 %H, 3.80 %N. Anal. Calcd. for $C_{144}Cu_{3}H_{116}N_{12}O_{64}Zr_{12}$ (**3**): 40.00 %C, 2.70 %H, 3.89 %N. Found: 46.37 %C, 3.10 %H, 3.96 %N. The large differences between the expected and observed elemental percentages can be attributed to the large number of defect sites within Zr-based frameworks, wherein many open coordination sites exist at the Zr nodes. These sites may be occupied by benzoic acid, acetate, or DMF molecules that are strongly bound to the nodes.[36,37] For PCN-224, the Zr nodes contain 12 potential coordination sites, six of which are coordinated to the porphyrinic ligand. Thus, for a unit of MOFs **1–3**, there exists 12 possible coordination sites for other molecular species. Calculations of the C, H, and N elemental percentages for **1–3** including benzoic acid and DMF molecules yielded values closer to the experimentally determined values.

Table S1 | Crystallographic data for the structure refinement of **3**.

Empirical Formula	$C_{10.67}H_{5.33}Cu_{0.22}N_{0.89}O_{3.85}Zr_{0.89}$
Formula Weight	302.77 g/mol
Temperature	100(2) K
Radiation	CuKα (λ = 1.54178 Å)
Crystal System	Cubic
Space Group	*Im*–3*m*
Unit Cell Dimensions	a = 38.4520(7) Å, α = 90°<br>b = 38.4520(7) Å, β = 90°<br>c = 38.4520(7) Å, γ = 90°
Volume	56853(3) $Å^3$
Z	54
Density (calculated)	0.478 g/cm^3
Absorption coefficient	2.047 mm^{-1}
F_{000}	8012.0
Crystal color	Red
Crystal size	0.02 × 0.02 × 0.02 mm^3
2θ range	3.24 to 136.74°
Index ranges	$-43 \le h \le 27$<br>$-41 \le k \le 46$<br>$-33 \le l \le 39$
Reflections collected	53006
Independent reflections	4827 [R_{int} = 0.0857]
Completeness to 2θ = 136.74°	98.77 %
Absorption correction	Multi-scan
Maximum and minimum transmission	0.7531 and 0.5815
Refinement method	Full-matrix least squares on F^2
Data / restraints / parameters	4827 / 0 / 95
Goodness-of-fit on F^{2a}	1.088
Final R indices [I > 2σ(I) = 7637 data][b]	R_1 = 0.0791, wR_2 = 0.2411
R indices (all data, Å)	R_1 = 0.0896, wR_2 = 0.2587
Largest diff. peak and hole	0.85 and –0.51 e.$Å^{-3}$

[a]GooF = $[\Sigma[w(F_o^2-F_c^2)^2] / (n-p)]^{1/2}$ where n is the number of reflections and p is the total number of parameters refined.

[b]$R_1 = \Sigma||F_o|-|F_c|| / \Sigma|F_o|$; $wR_2 = [\Sigma[w(F_o^2-F_c^2)^2] / \Sigma[w(F_o^2)^2]]^{1/2}$

Table S2 | Spin Hamiltonian parameters of the CW EPR spectra for **1–3** measured at 80 K.

Parameter	**1**	**2***	**3***
$g_{\|\|}$	2.186	2.186	2.186
$g_{\perp}$	2.042	2.042	2.042
$A_{\|\|}^{Cu}$	611	611	611
$A_{\perp}^{Cu}$	79.4	79.4	79.4
$A_{\|\|}^{N}$	43.2	43.2	43.2
$A_{\perp}^{N}$	48.2	48.2	48.2
Γ_G*	0.59	0.59	0.59
Γ_L**	0.20	0.83	1.53

* Gaussian linewidth of the CW EPR spectra.
** Lorentzian linewidth of the CW EPR spectra.

Table S3 | T_2 relaxation times and fit parameters for **1** at the 2942 G and 3328 G resonances. Echo decay curves were fitted with the monoexponential function: $\boldsymbol{I(2\tau) = Ae^{(2\tau/T_2)} + C}$.

T (K)	2942 G	3328 G
	T_2 (μs)	T_2 (μs)
10	0.636(6)	0.613(2)
15	0.626(5)	0.550(2)
20	0.585(3)	0.512(2)
30	0.466(2)	0.439(1)
40	0.354(2)	0.371(2)
60	0.232(2)	0.240(2)
80	0.148(1)	0.151(1)

Table S4 | T_2 relaxation times and fit parameters for **2** at the 2942 G and 3328 G resonances. Echo decay curves were fitted with the monoexponential function: $\boldsymbol{I(2\tau) = Ae^{(2\tau/T_2)} + C}$.

T (K)	2942 G	3328 G
	T_2 (μs)	T_2 (μs)
10	0.187(1)	0.123(1)
15	0.177(1)	0.137(1)
20	0.172(1)	0.125(1)
30	0.157(1)	0.114(1)
40	0.138(1)	0.104(1)
60	0.0923(9)	0.0779(7)
80	0.0542(5)	0.0506(4)

Table S5 | T_2 relaxation times and fit parameters for **3** at the 3010 G and 3410 G resonances. Echo decay curves were fitted with the monoexponential function: $\boldsymbol{I(2\tau) = Ae^{(2\tau/T_2)} + C}$.

T (K)	3010 G	3410 G
	T_2 (μs)	T_2 (μs)
10	0.0584(4)	0.0478(4)
15	0.0580(4)	0.0473(3)
20	0.0576(5)	0.0473(3)
30	0.0540(4)	0.0460(4)
40	0.0479(5)	0.0434(5)
60	0.0383(5)	0.0349(4)
80	0.0268(10)	0.0239(3)

Table S6 | T_1 relaxation times and fit parameters for **1** at the 2942 G and 3328 G resonances. Saturation recovery curves were fitted with the exponential function including spectral diffusion contributions: $\boldsymbol{I(\tau) = (A_0 - A_1)e^{-\tau/T_1 - \sqrt{\tau/a}} + A_1}$.

T (K)	2942 G		3328 G	
	T_1 (s)	a (s)	T_1 (s)	a (s)
10	5.40(8) × 10^{-4}	3.7(7) × 10^{-2}	4.6(1) × 10^{-4}	8(2) × 10^{-3}
15	2.68(4) × 10^{-4}	2.4(5) × 10^{-3}	2.65(5) × 10^{-4}	8(2) × 10^{-3}
20	1.49(3) × 10^{-4}	8(2) × 10^{-3}	1.47(3) × 10^{-4}	4.5(8) × 10^{-3}
30	5.8(2) × 10^{-5}	3(1) × 10^{-3}	6.0(2) × 10^{-5}	2.1(7) × 10^{-3}
40	2.76(9) × 10^{-5}	1.6(7) × 10^{-3}	2.7(1) × 10^{-5}	1.1(6) × 10^{-3}
60	7.1(2) × 10^{-6}	8(5) × 10^{-4}	6.3(3) × 10^{-6}	2(1) × 10^{-4}
80	2.9(1) × 10^{-6}	4(2) × 10^{-5}	1.92(7) × 10^{-6}	3(1) × 10^{-5}

Table S7 | T_1 relaxation times and fit parameters for **1** at the 2942 G and 3328 G resonances. Saturation recovery curves were fitted with the stretched exponential function: $\boldsymbol{I(\tau) = 1 - A_0 e^{-(\tau/T_1)^{\beta}} + C}$.

T (K)	2942 G		3328 G	
	T_1 (s)	β	T_1 (s)	β
10	4.81(2) × 10^{-4}	0.923(4)	3.72(3) × 10^{-4}	0.869(8)
15	2.42(1) × 10^{-4}	0.934(4)	2.22(1) × 10^{-4}	0.895(4)
20	1.31(1) × 10^{-4}	0.920(6)	1.24(1) × 10^{-4}	0.897(4)
30	5.10(5) × 10^{-5}	0.92(1)	5.13(6) × 10^{-5}	0.90(1)
40	2.44(3) × 10^{-5}	0.93(1)	2.32(3) × 10^{-5}	0.91(2)
60	6.52(5) × 10^{-6}	0.95(1)	5.32(8) × 10^{-6}	0.92(2)
80	2.31(3) × 10^{-6}	0.90(2)	1.56(1) × 10^{-6}	0.91(1)

Table S8 | T_1 relaxation times and fit parameters for **2** at the 2942 G and 3328 G resonances. Saturation recovery curves were fitted with the exponential function including spectral diffusion contributions: $\boldsymbol{I(\tau) = (A_0 - A_1)e^{-\tau/T_1 - \sqrt{\tau/a}} + A_1}$.

	2942 G		3328 G	
T (K)	T_1 (s)	a (s)	T_1 (s)	a (s)
10	3.04(6) × 10^{-4}	2.0(2) × 10^{-3}	4.2(4) × 10^{-4}	3.5(3) × 10^{-4}
15	1.97(4) × 10^{-4}	1.2(1) × 10^{-3}	2.0(1) × 10^{-4}	3.4(4) × 10^{-4}
20	1.26(3) × 10^{-4}	8.4(9) × 10^{-4}	8.5(4) × 10^{-5}	2.9(4) × 10^{-4}
30	5.6(2) × 10^{-5}	4.3(8) × 10^{-4}	3.66(9) × 10^{-5}	4.5(7) × 10^{-4}
40	2.8(2) × 10^{-5}	2.3(7) × 10^{-4}	1.82(4) × 10^{-5}	4.9(9) × 10^{-4}
60	6.8(2) × 10^{-6}	1.2(2) × 10^{-4}	5.03(9) × 10^{-6}	1.3(2) × 10^{-4}
80	3.1(2) × 10^{-6}	9(2) × 10^{-6}	1.78(5) × 10^{-6}	1.1(2) × 10^{-5}

Table S9 | T_1 relaxation times and fit parameters for **2** at the 2942 G and 3328 G resonances. Saturation recovery curves were fitted with the stretched exponential function: $\boldsymbol{I(\tau) = 1 - A_0 e^{-(\tau/T_1)^{\beta}} + C}$.

	2942 G		3328 G	
T (K)	T_1 (s)	β	T_1 (s)	β
10	2.07(2) × 10^{-4}	0.821(7)	1.48(3) × 10^{-4}	0.670(7)
15	1.33(1) × 10^{-4}	0.816(4)	9.58(8) × 10^{-5}	0.723(6)
20	8.63(4) × 10^{-5}	0.822(4)	5.05(5) × 10^{-5}	0.779(8)
30	3.96(4) × 10^{-5}	0.83(1)	2.80(1) × 10^{-5}	0.866(4)
40	2.00(4) × 10^{-5}	0.84(2)	1.52(1) × 10^{-5}	0.908(6)
60	5.48(5) × 10^{-6}	0.900(5)	4.19(2) × 10^{-6}	0.917(5)
80	1.84(3) × 10^{-6}	0.82(2)	1.26(1) × 10^{-6}	0.873(6)

Table S10 | T_1 relaxation times and fit parameters for **3** at the 3010 G and 3410 G resonances. Saturation recovery curves were fitted with the exponential function including spectral diffusion contributions: $\boldsymbol{I(\tau) = (A_0 - A_1)e^{-\tau/T_1 - \sqrt{\tau/a}} + A_1}$.

T (K)	3010 G		3410 G	
	T_1 (s)	a (s)	T_1 (s)	a (s)
10	$3.2(1) \times 10^{-4}$	$2.25(9) \times 10^{-4}$	$9(1) \times 10^{-5}$	$5.5(8) \times 10^{-5}$
15	$1.83(6) \times 10^{-4}$	$2.06(9) \times 10^{-4}$	$5.8(6) \times 10^{-5}$	$5.9(8) \times 10^{-5}$
20	$1.35(5) \times 10^{-4}$	$1.8(1) \times 10^{-4}$	$5.7(5) \times 10^{-5}$	$7(1) \times 10^{-5}$
30	$5.9(3) \times 10^{-5}$	$1.2(1) \times 10^{-4}$	$4.1(4) \times 10^{-5}$	$4.1(6) \times 10^{-5}$
40	$2.9(2) \times 10^{-5}$	$8(1) \times 10^{-5}$	$2.0(2) \times 10^{-5}$	$3.7(6) \times 10^{-5}$
60	$8.5(6) \times 10^{-6}$	$1.7(3) \times 10^{-5}$	$4.6(2) \times 10^{-6}$	$1.8(3) \times 10^{-5}$
80	$2.8(7) \times 10^{-6}$	$3(2) \times 10^{-6}$	$1.6(1) \times 10^{-6}$	$4.0(9) \times 10^{-6}$

Table S11 | T_1 relaxation times and fit parameters for **3** at the 3010 G and 3410 G resonances. Saturation recovery curves were fitted with the stretched exponential function: $\boldsymbol{I(\tau) = 1 - A_0 e^{-(\tau/T_1)^{\beta}} + C}$.

T (K)	3010 G		3410 G	
	T_1 (s)	β	T_1 (s)	β
10	$1.04(1) \times 10^{-4}$	0.658(4)	$2.88(7) \times 10^{-5}$	0.66(1)
15	$7.43(6) \times 10^{-5}$	0.697(5)	$2.49(4) \times 10^{-5}$	0.71(1)
20	$5.83(3) \times 10^{-5}$	0.711(4)	$2.40(4) \times 10^{-5}$	0.71(1)
30	$3.05(3) \times 10^{-5}$	0.749(6)	$1.61(3) \times 10^{-5}$	0.70(1)
40	$1.62(2) \times 10^{-5}$	0.77(1)	$1.01(2) \times 10^{-5}$	0.75(1)
60	$4.45(8) \times 10^{-6}$	0.78(2)	$2.92(2) \times 10^{-6}$	0.829(8)
80	$1.25(9) \times 10^{-6}$	0.75(6)	$9.5(2) \times 10^{-7}$	0.83(2)

Table S12 | Direct, Raman and Orbach-Aminov contributions to T_1 of **1–3** at 2942/3010 G from fits to the temperature dependence of T_1 obtained from pulsed EPR.

Compound	A_{Dir} (K^{-1} s^{-1})	A_{Raman} (s^{-1})	A_{Orb} (K^{-2} s^{-1})	θ_D (K)	θ_{Orb} (K)
**1**	103	8.05×10^7	0.181	294	9.92×10^{-4}
**2**	103	8.05×10^7	0.275	294	9.92×10^{-4}
**3**	103	8.05×10^7	0.263	294	9.92×10^{-4}

Table S13 | Direct, Raman and Orbach-Aminov contributions to T_1 of **1–3** at 3328/3410 G from fits to the temperature dependence of T_1 obtained from pulsed EPR.

Compound	A_{Dir} (K^{-1} s^{-1})	A_{Raman} (s^{-1})	A_{Orb} (K^{-2} s^{-1})	θ_D (K)	θ_{Orb} (K)
**1**	103	8.05×10^7	0.166	269	9.92×10^{-4}
**2**	103	8.05×10^7	0.301	269	9.92×10^{-4}
**3**	103	8.05×10^7	0.800	269	9.92×10^{-4}

Table S14 | Fit parameters for the temperature dependence fits to the Hahn echo decay of **1–3** across both resonances. Details are described in the EPR section above.

Cu–Cu Distance (Å)	Number of Distances	m_i	f_i
13.595	4	4	0.7
19.226	2	2	1.0
23.547	8	8	0.6
27.190	4	4	0.7
30.399	8	8	0.8
33.300	8	8	0.6
35.969	16	16	0.8
38.452	6	6	0.7
40.785	12	12	0.5
42.991	8	8	0.4
45.089	8	8	0.07
47.094	8	8	0.01
49.016	14	14	5
49.018	10	10	0.004
52.648	16	16	7
54.379	12	12	4
56.053	16	16	8
57.678	10	10	0.8
59.259	24	24	12

Table S15│ Cole-Cole fit parameters for variable temperature ac susceptibility data collect of **3** at 2940 G.

T (K)	X_T (emu/mol)	X_S (emu/mol)	α	τ (s)
5	0.745	0	2.86×10^{-2}	6.69×10^{-4}
6	0.626	0	4.13×10^{-2}	6.88×10^{-4}
7	0.533	0	2.79×10^{-2}	6.83×10^{-4}
8	0.464	0	1.62×10^{-2}	6.30×10^{-4}
9	0.413	0	2.22×10^{-2}	5.54×10^{-4}
10	0.374	0	2.38×10^{-2}	4.65×10^{-4}

Table S16│ Cole-Cole fit parameters for variable temperature ac susceptibility data collect of **3** at 3410 G.

T (K)	X_T (emu/mol)	X_S (emu/mol)	α	τ (s)
5	0.745	0	5.41×10^{-3}	6.82×10^{-4}
6	0.623	0	1.41×10^{-2}	6.70×10^{-4}
7	0.531	0	0	6.26×10^{-4}
8	0.463	0	0	5.69×10^{-4}
9	0.415	0	0	5.16×10^{-4}
10	0.373	0	0	4.46×10^{-4}

Table S17│ Cole-Cole fit parameters for variable field AC susceptibility data collect of **3** at 5 K.

B (T)	X_T (emu/mol)	X_S (emu/mol)	α	τ (s)
0.025	0.762	0.353	4.05×10^{-2}	3.27×10^{-4}
0.050	0.754	0.143	1.57×10^{-2}	4.61×10^{-4}
0.075	0.760	7.47×10^{-2}	3.48×10^{-2}	5.82×10^{-4}
0.1	0.754	4.79×10^{-2}	3.05×10^{-2}	6.41×10^{-4}
0.2	0.765	0	6.56×10^{-2}	7.17×10^{-4}
0.3	0.748	0	2.58×10^{-2}	7.01×10^{-4}
0.4	0.744	0	1.40×10^{-2}	6.54×10^{-4}
0.5	0.743	0	1.30×10^{-2}	5.77×10^{-4}
0.6	0.743	0	1.60×10^{-2}	5.01×10^{-4}
0.7	0.740	0	2.93×10^{-2}	4.47×10^{-4}
0.8	0.735	0	1.58×10^{-2}	3.84×10^{-4}
0.9	0.733	0	2.77×10^{-2}	3.48×10^{-4}
1.0	0.740	0	3.39×10^{-2}	3.20×10^{-4}
1.2	0.727	0	3.77×10^{-2}	2.58×10^{-4}
1.4	0.728	0	4.36×10^{-2}	2.23×10^{-4}
1.6	0.698	0	2.82×10^{-2}	1.93×10^{-4}
1.8	0.700	0	1.24×10^{-2}	1.57×10^{-4}
2.0	0.702	0	3.94×10^{-2}	1.43×10^{-4}

Table S18 | Fit parameters for the field dependence of τ extracted from fits to ac susceptibility curves.

Compound	c (T^{-4} s^{-1})*	d (s^{-1})	e (T^{-2})	f (T^{-2})	g (s^{-1})	h (T^{-2})
**3**	8(103)	3.8(2) × 10^3	2.1(2) × 10^2	7(1) × 10^2	10(2)	0.2(1)

*The large error in c may be due to overparametrization of the fit function. Nonetheless, it is the magnitude of the value that is of interest for a qualitative understanding on the magnitude of the direct process as a function of applied field.

Table S19 | Fit parameters of the linear fits to the $1/[v(P_0/P–1)]$ vs P/P_0 plots to calculate BET surface areas of **1–3**. Maximum P/P_0 values determined from the $v(P_0–P)$ vs P/P_0 plots are also included.

Compound	**1**	**2**	**3**
Maximum P/P_0	0.121	0.123	0.121
Slope	1.31 × 10^{-3}	1.63 × 10^{-3}	1.28 × 10^{-3}
Intercept	1.55 × 10^{-5}	1.89 × 10^{-5}	1.60 × 10^{-5}
R^2	0.99794	0.99735	0.99777
BET Surface Area (m^2/g)	3298	2641	3365

Table S20 | Variable-power nutation frequencies of **1–3** collected at the 2942 G resonance. The frequencies tabulated are those with the maximum amplitude in the Fourier transform that does not correspond to a nuclear Larmor frequency. The pulsed magnetic field B_1 is calculated relative to its value at the lowest microwave power (17 dB) with the equation: $\boldsymbol{B_1} = \sqrt{\mathbf{10^{-0.1A}}/\mathbf{10^{-1.70}}}$ where A is the attenuation in dB.

Relative B_1	2.51		1.99		1.26		1	
	Ω_R (MHz)	A (dB)	Ω_R (MHz)	A (dB)	Ω_R (MHz)	A (dB)	Ω_R (MHz)	A (dB)
**1**	16.9	9	13.9	11	9.0	15	7.7	17
**2**	17.7	9	14.9	11	10.2	15	8.1	17
**3**	19.6	9	15.9	11	10.0	15	8.1	17

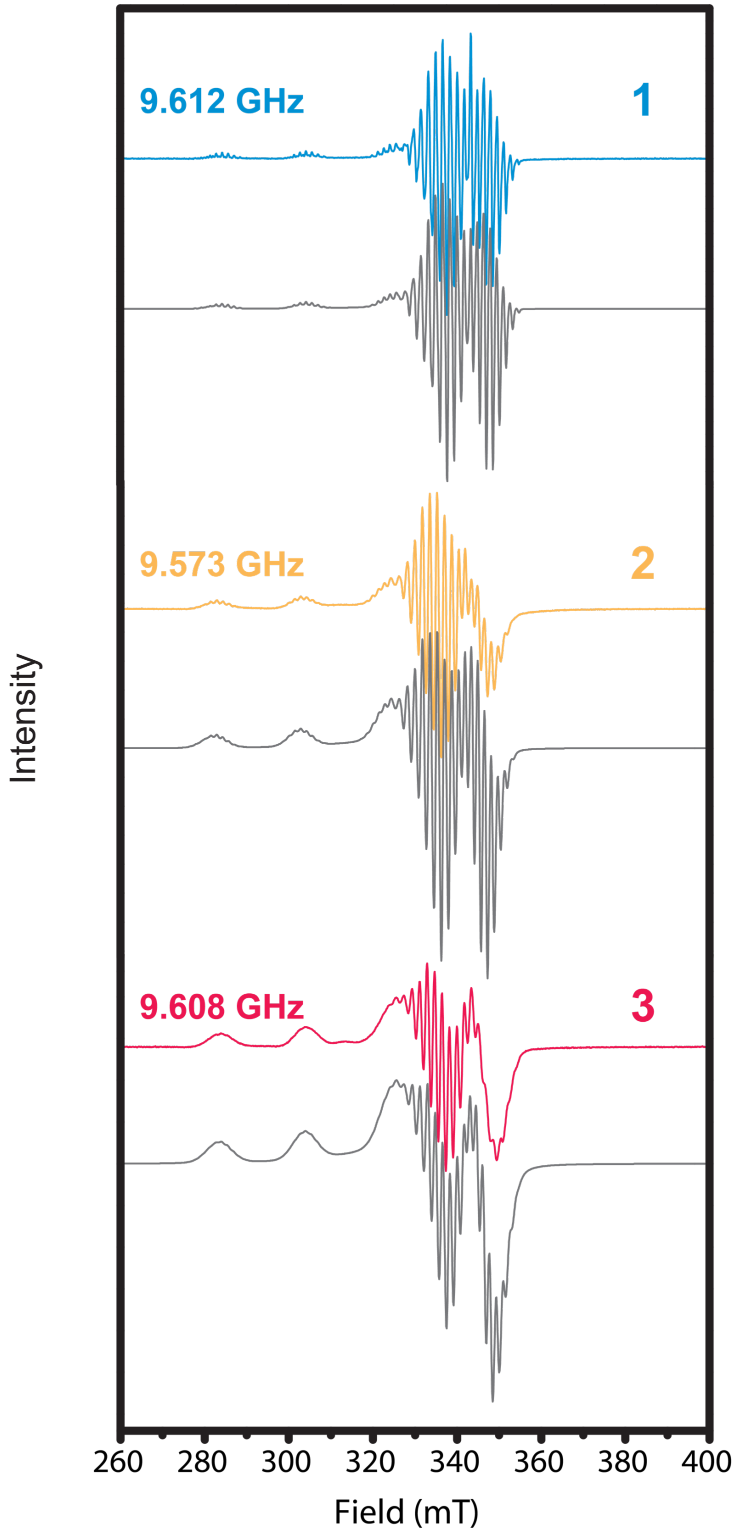

Figure S1│Continuous wave (cw) EPR spectra of **1**–**3** collected at 80 K. Colored lines represent experimental data, and the fitted spectra are shown in the gray lines. Parameters of the fitted spectra are reported in Table S2.

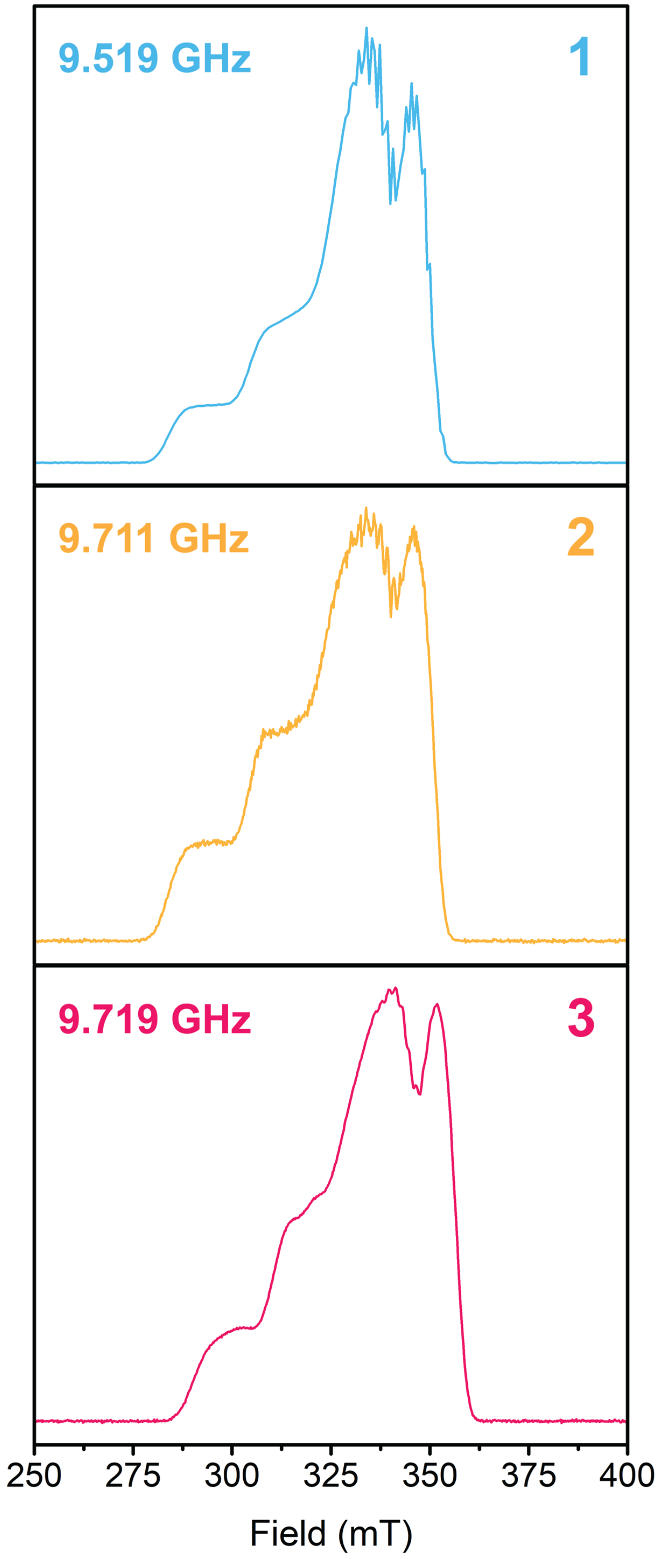

Figure S2 | Echo-detected field-swept X-band EPR spectra of **1**–**3** collected at 20 K. Frequencies for each measurement are included in the figure.

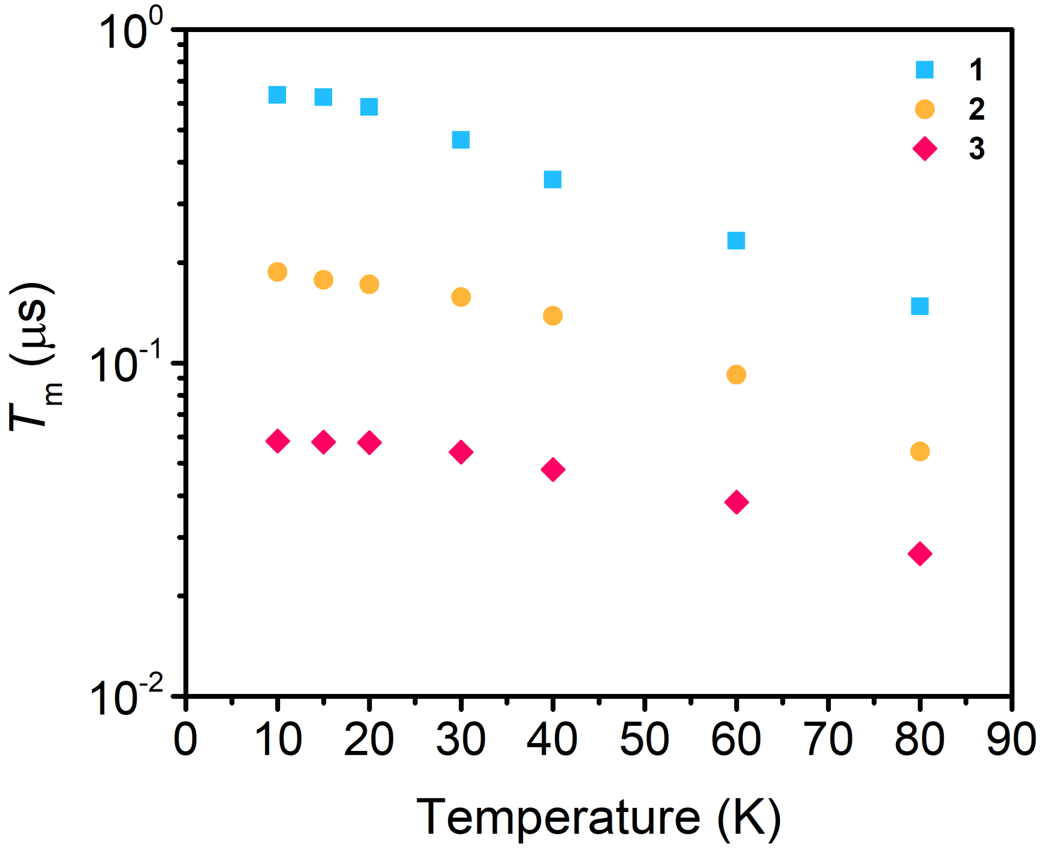

Figure S3│Variable-temperature coherence times (T_m) of **1**–**3** measured at 2942 G for **1** and **2** and at 3010 G for **3**.

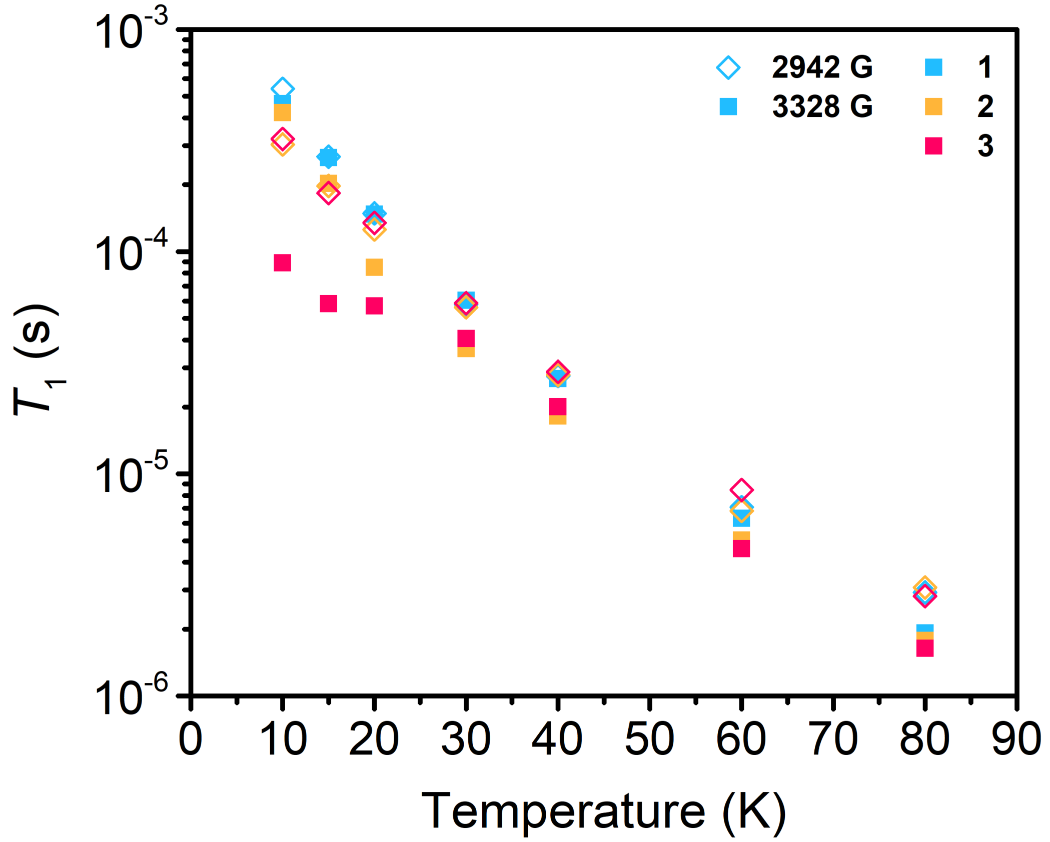

Figure S4 | T_1 values of **1–3** at 2942/3010 G (diamond) and 3328/3410 G (square) across 10–80 K.

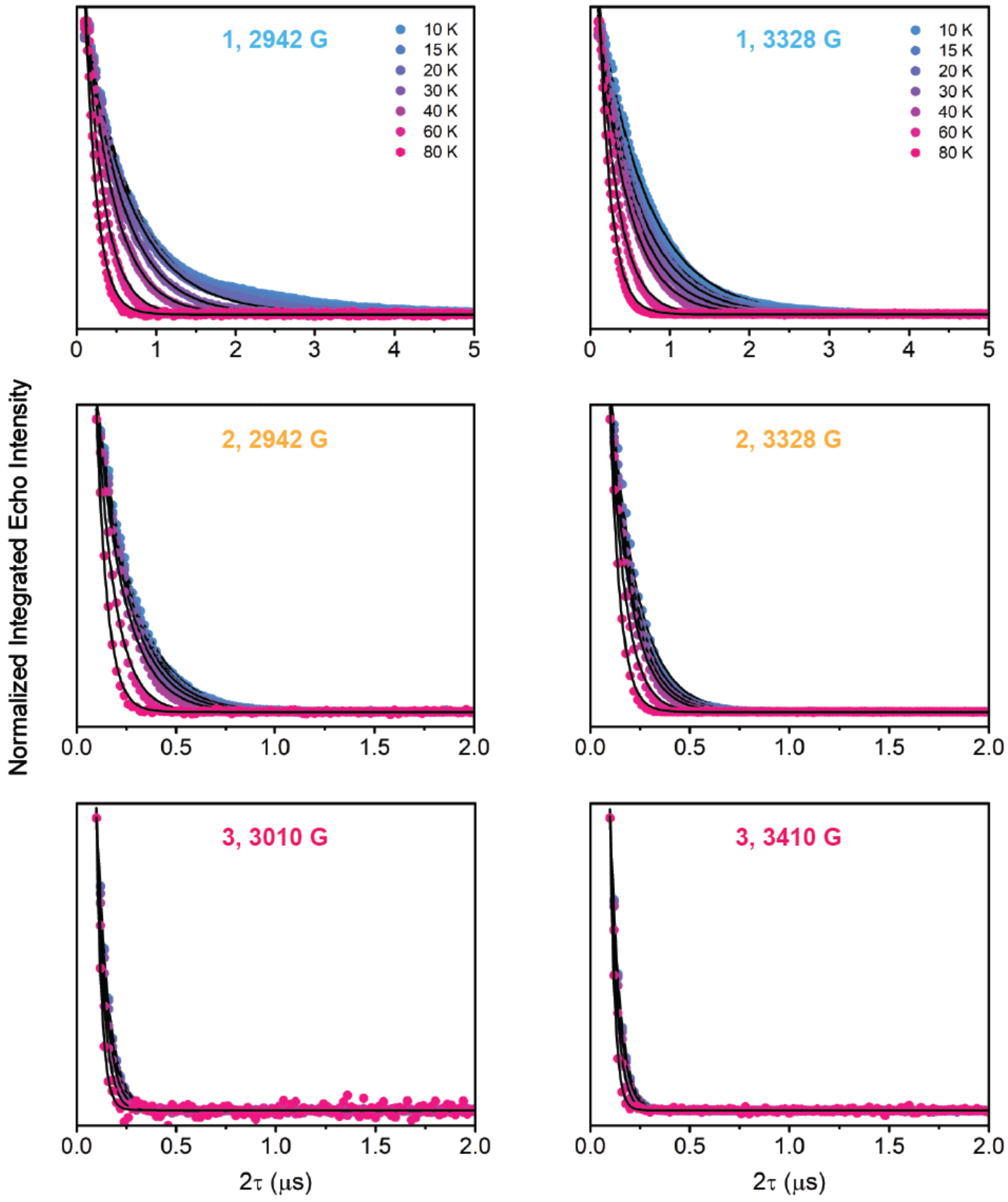

Figure S5 | Variable-temperature Hahn echo decay curves for **1–3** measured at 2942 and 3328 G for **1** and **2**, and at 3010 G and 3410 G for **3**. Black lines represent best fits to the exponential decays. Details of the fits are described in the EPR spectroscopy experimental section. T_2 values and fit parameters obtained are reported in Tables S3–S5.

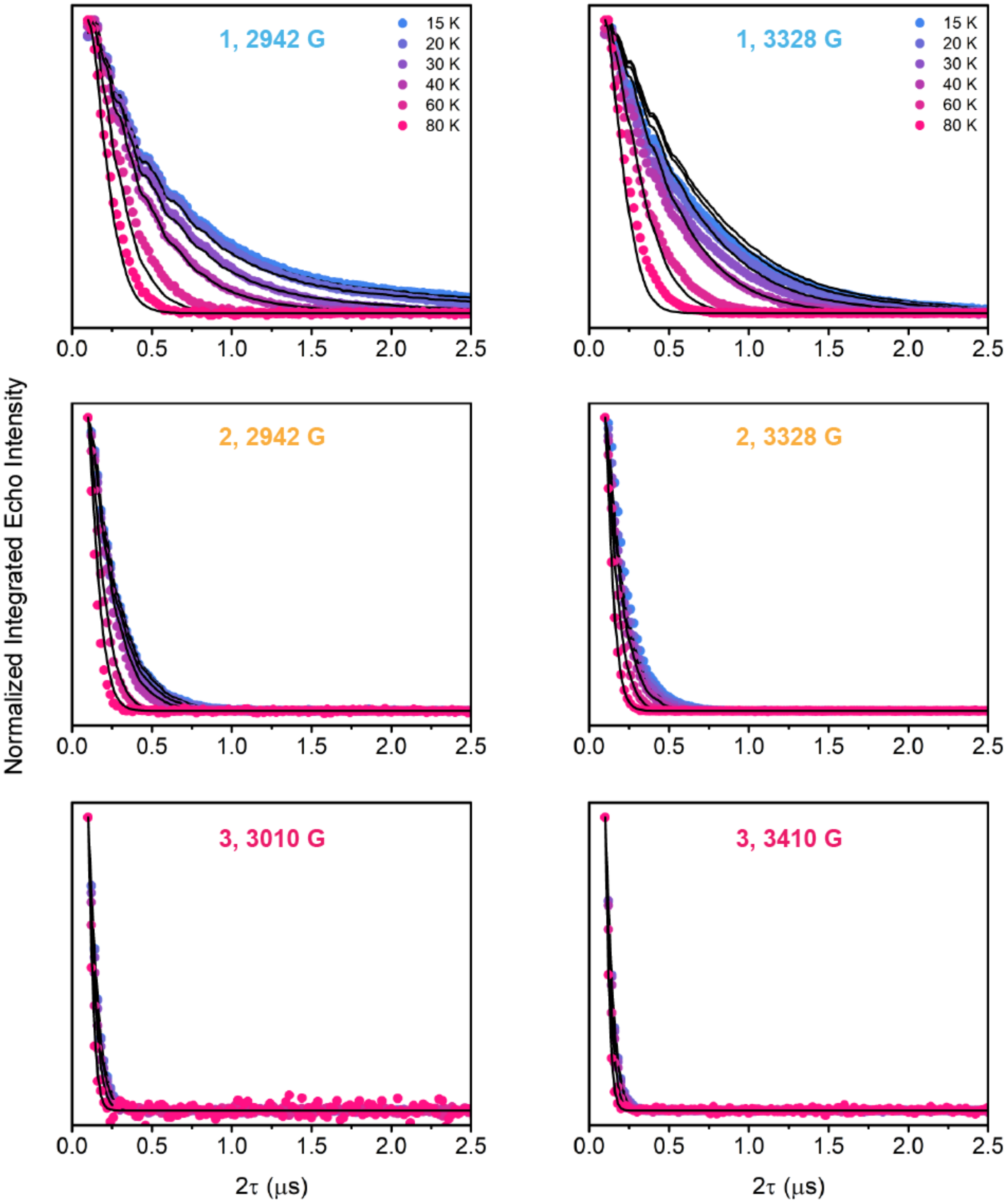

Figure S6 | Fits based on the model for spin-spin interactions to the variable-temperature Hahn echo decay curves for **1–3** measured at 2942 and 3328 G for **1** and **2**, and at 3010 G and 3410 G for **3**. Black lines represent best fits to the experimental data. Details of the fits are described in the EPR spectroscopy experimental section. Fit parameters are detailed in Table S13.

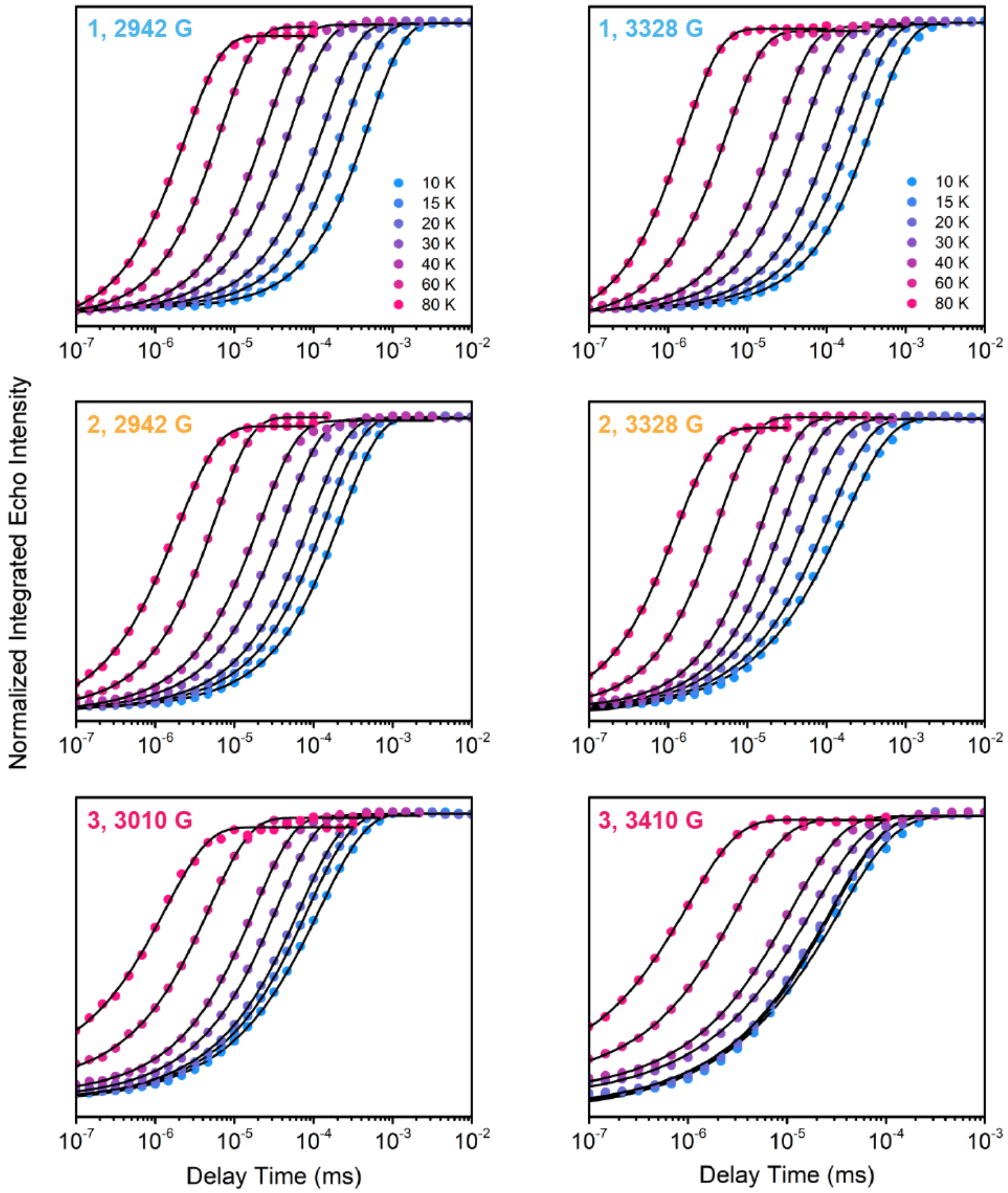

Figure S7 | Variable-temperature saturation recovery curves for **1–3** measured at 2942 and 3328 G for **1** and **2**, and at 3010 G and 3410 G for **3**. Black lines represent the fits accounting for spectral diffusion to the data. Details of the fits are described in the EPR spectroscopy experimental section. T_1 values and fit parameters obtained are reported in Tables S6, S8, and S10.

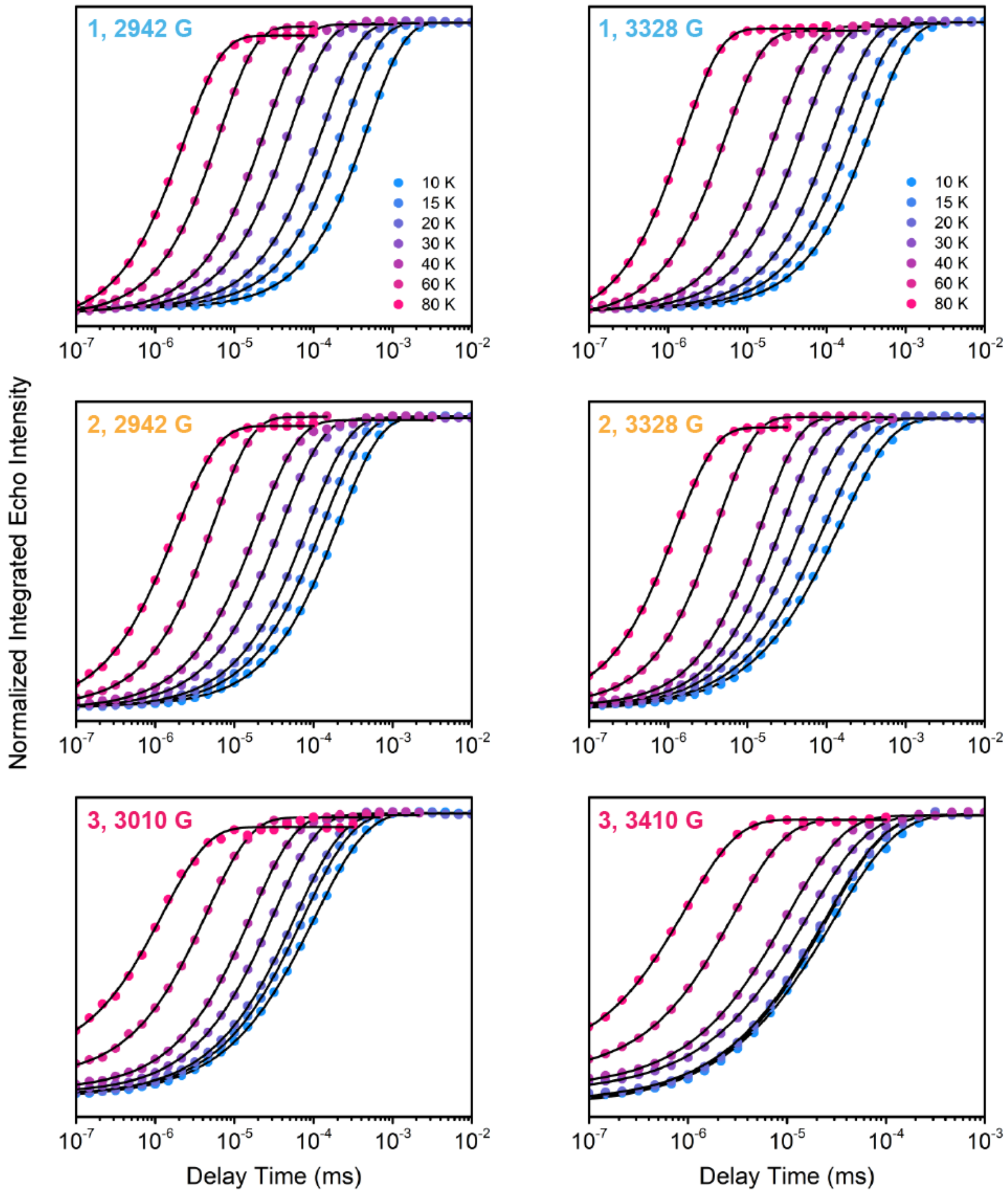

Figure S8 | Variable-temperature saturation recovery curves for **1–3** measured at 2942 and 3328 G for **1** and **2**, and at 3010 G and 3410 G for **3**. Black lines represent the stretched exponential fits to the data. Details of the fits are described in the EPR spectroscopy experimental section. T_1 values and fit parameters obtained are reported in Tables S7, S9, and S11.

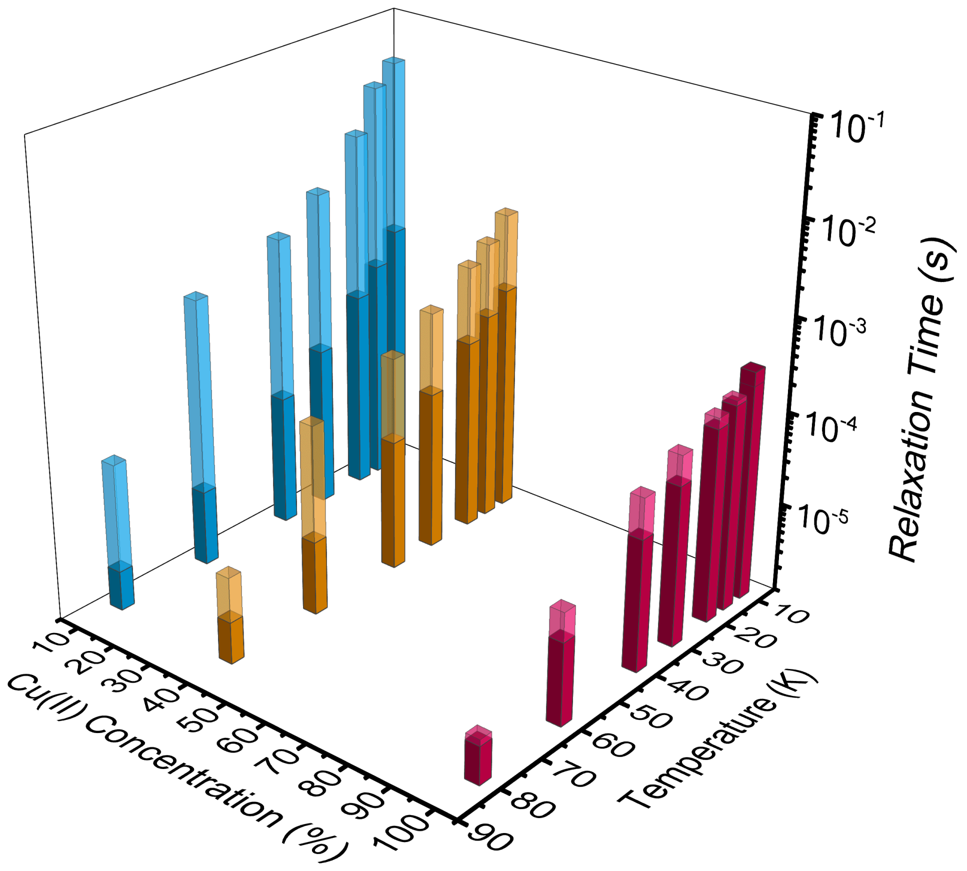

Figure S9 | Temperature dependence of *a* and T_1 collected at the 2942/3010 G resonance for **1**, **2**, and **3**. Solid bars represent the T_1 relaxation time, while the transparent bars represent relaxation time induced by spectral diffusion (*a*). Values for *a* and T_1 are reported in Tables S6, S8 and S10.

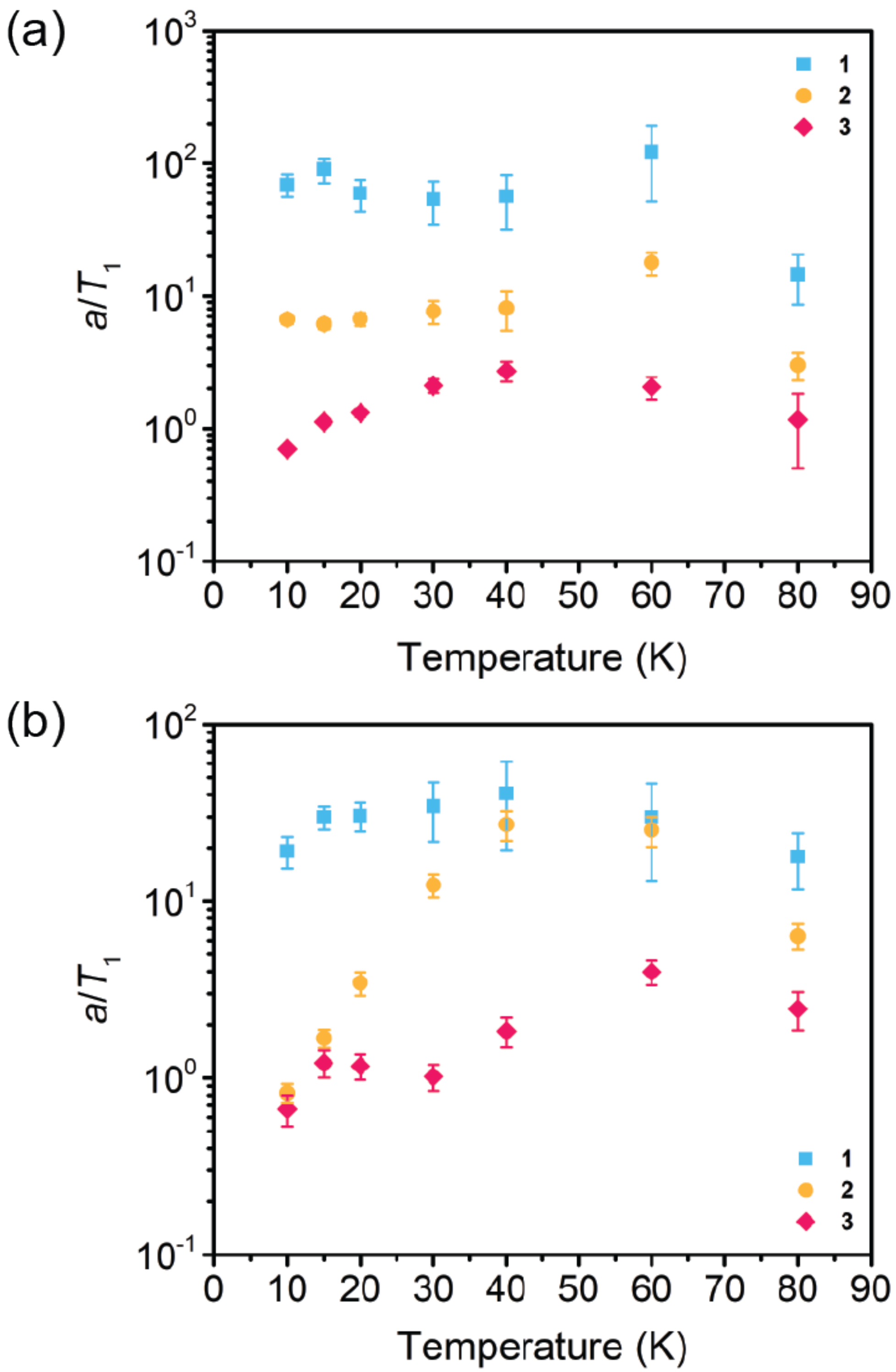

Figure S10 | Temperature dependence of a/T_1 of **1–3** collected at (a) 2942/3010 G and (b) 3328/3410 G resonance. Error bars are included.

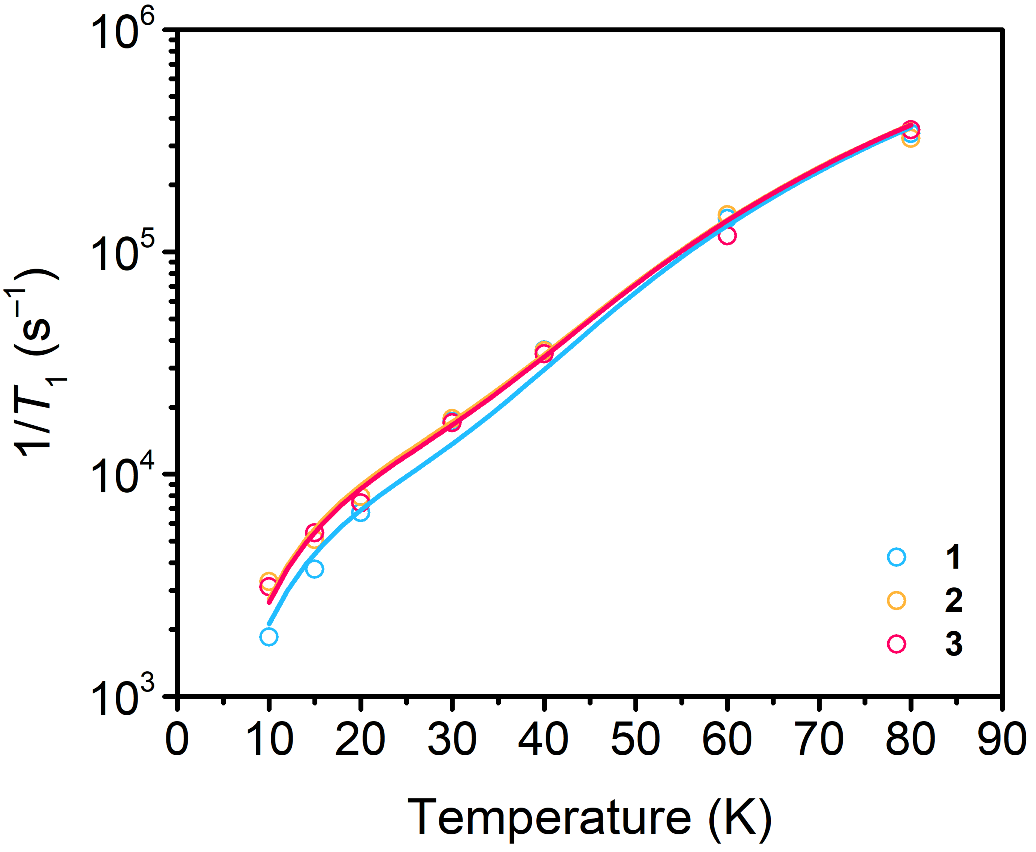

Figure S11 | Fits to the T_1 temperature dependence for **1**, **2**, and **3** at 2942 G. Results of the fits are reported in Table S12.

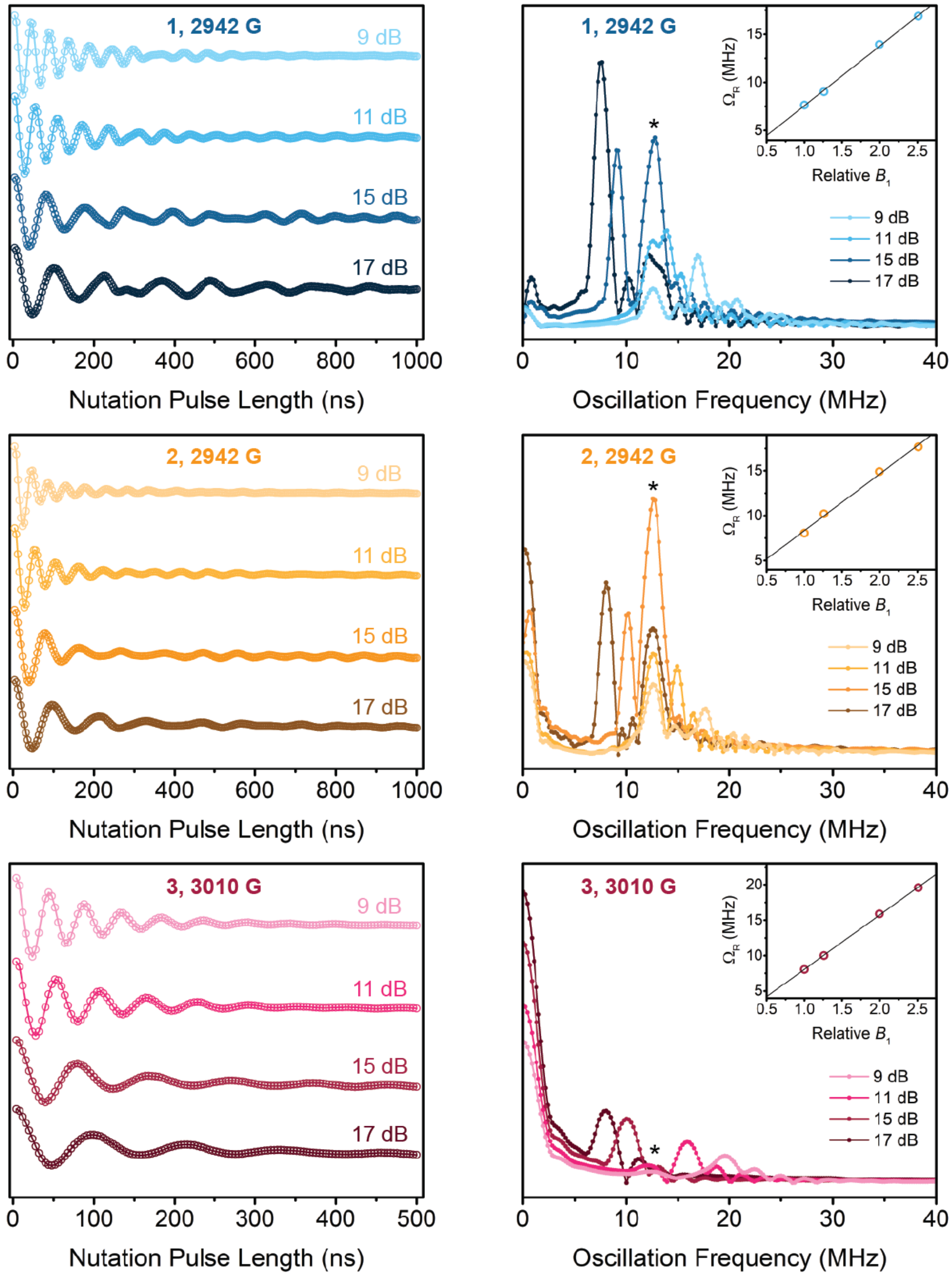

Figure S12 | Variable-power nutation data of **1–3** collected at the 2942/3010 G resonance at 20 K are shown on the left. Fourier transforms of the data are shown on the right. Asterisks in the plots of the Fourier transform indicate peaks corresponding to the Larmor frequency of ^{1}H at the measured fields (12.53 MHz at 2942 G, 12.82 MHz at 3010 G). Insets display Rabi frequencies (Ω_R) as a function of B_1. B_1 values are calculated relative to 17 dB, with the equation: $\boldsymbol{B_1} = \sqrt{\mathbf{10^{-0.1\mathit{A}}}/\mathbf{10^{-1.70}}}$ where A is the attenuation in dB. Linear fits to Ω_R are also shown.

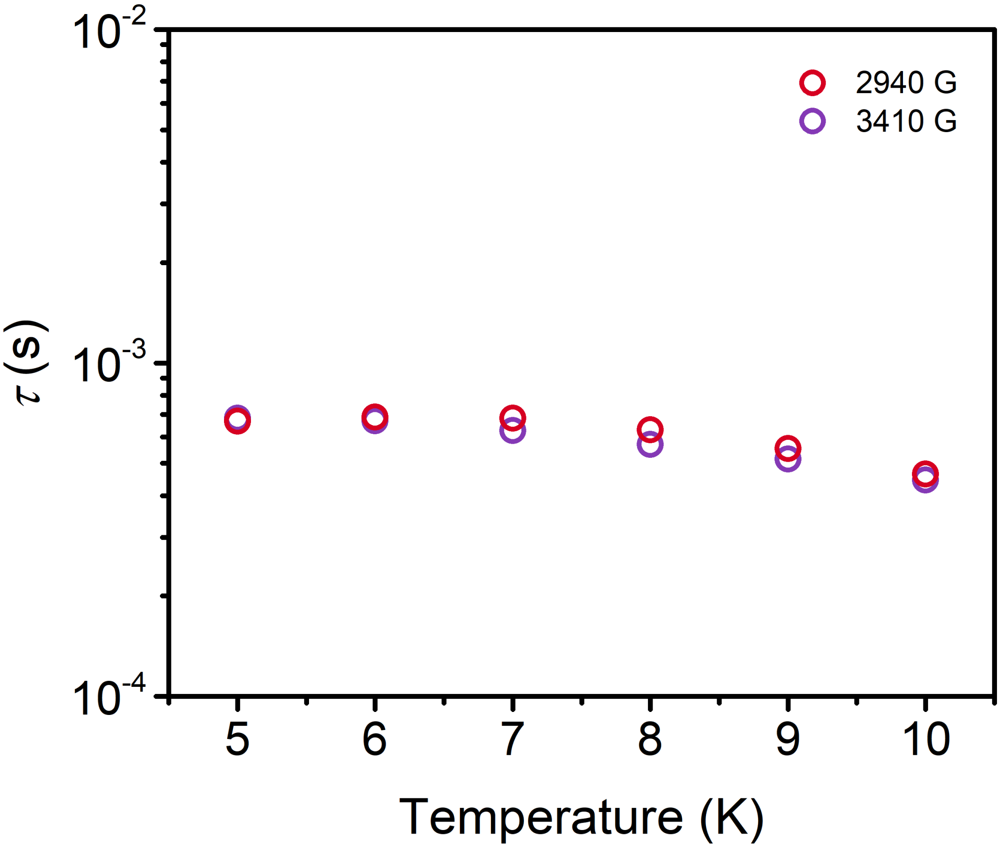

Figure S13│ Temperature dependence of τ extracted from ac susceptibility measurements of **3** at 2940 G and 3410 G.

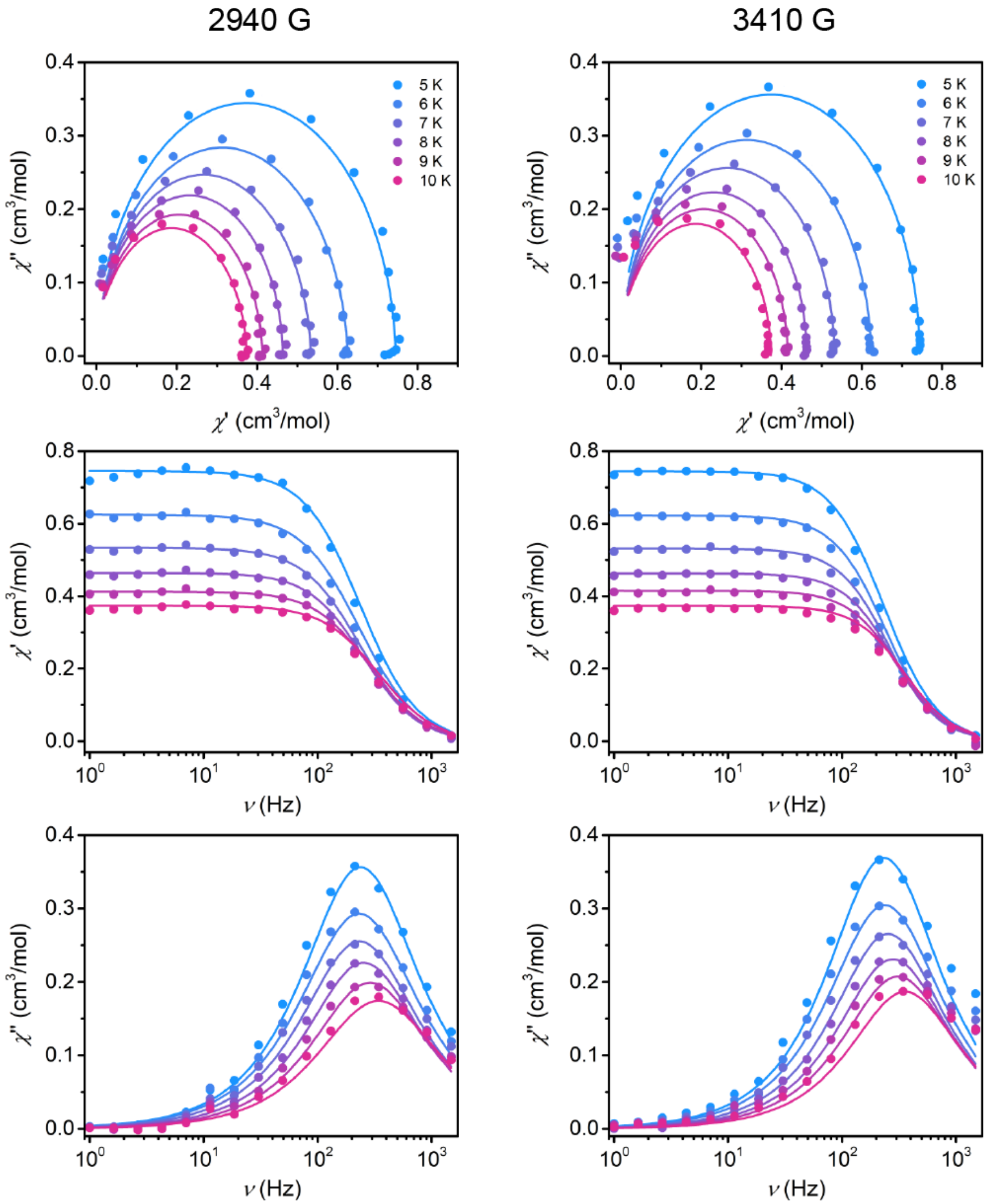

Figure S14 | (Top) Cole-Cole plots of the real component χ' and the imaginary component χ'' of **3** across 5–10 K. Frequency dependence of the real component χ' (middle) and the imaginary component χ'' (bottom) as a function of temperature from 5–10 K. Solid lines represent best fits to the data with the generalized Debye model.

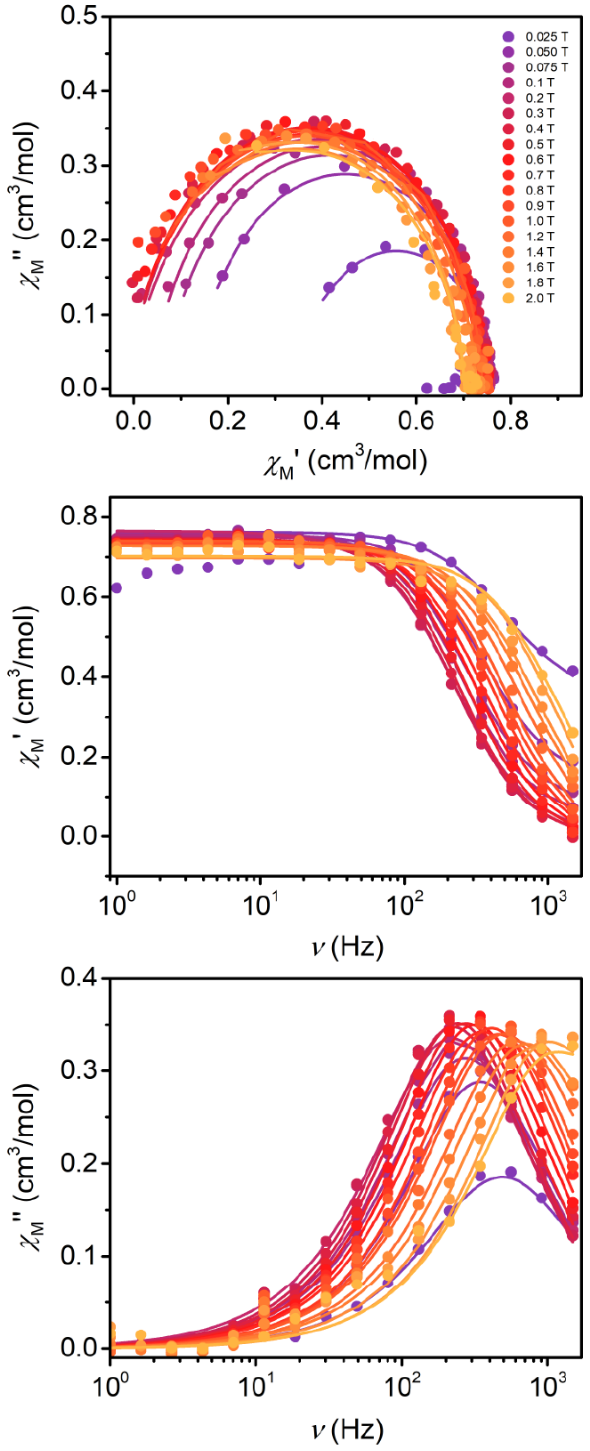

Figure S15 | (Top) Cole-Cole plots of the real component χ' and the imaginary component χ'' of **3** across 0.025–2 T at 5 K. Frequency dependence of the real component χ' (middle) and the imaginary component χ'' (bottom) as a function of magnetic field from 0.025–2 T at 5 K. Solid lines represent best fits to the data with the generalized Debye model.

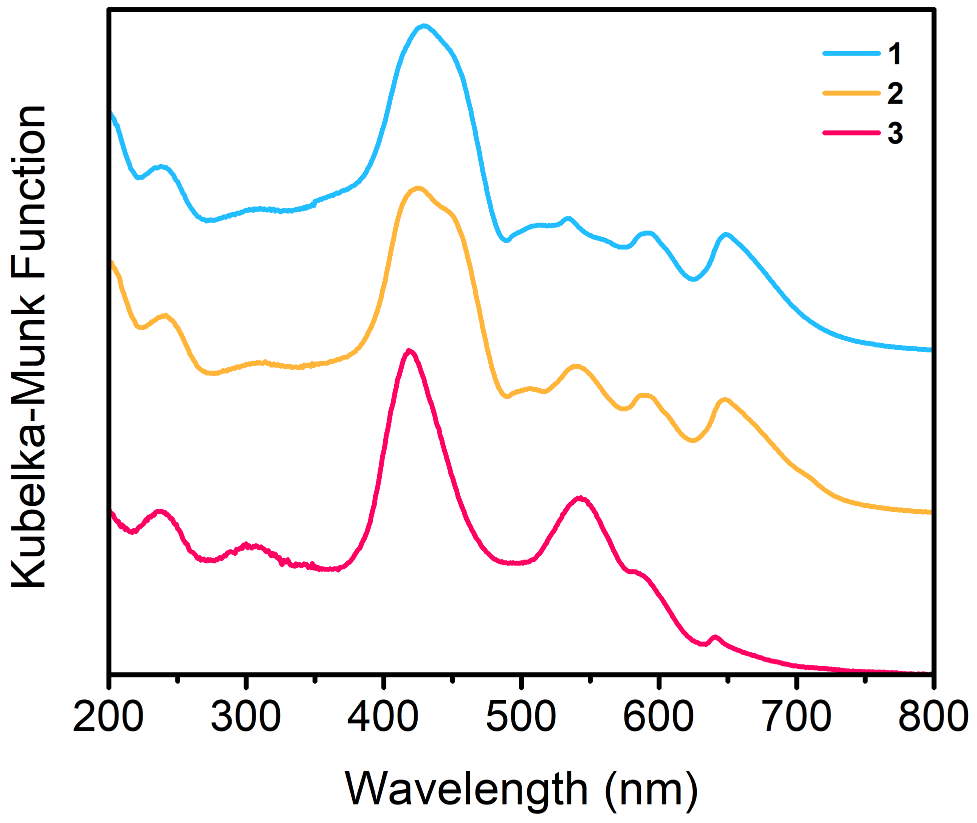

Figure S16 | Diffuse-reflectance UV-Vis spectra for **1–3** measured in a 1:7 sample:$BaSO_4$ homogenous mixture.

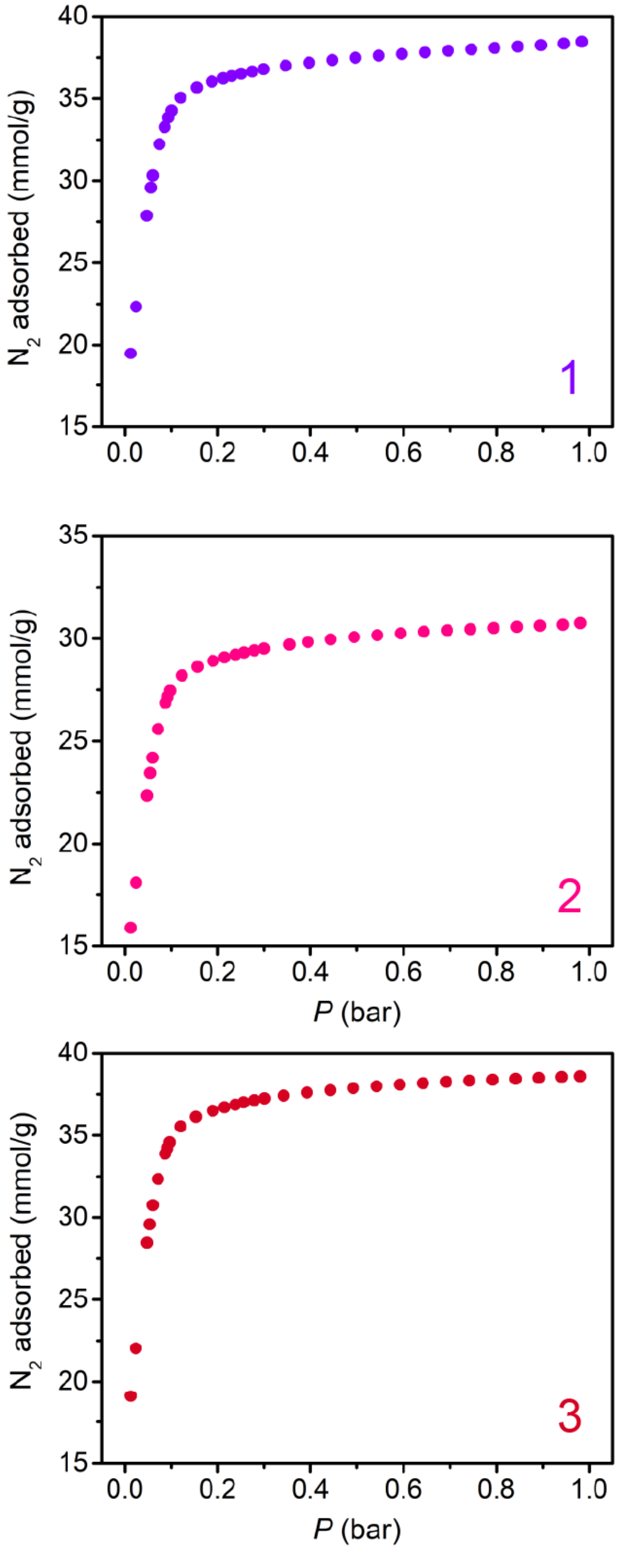

Figure S17 | N_2 adsorption isotherms at 77 K used to calculate BET surface areas of **1**–**3**.

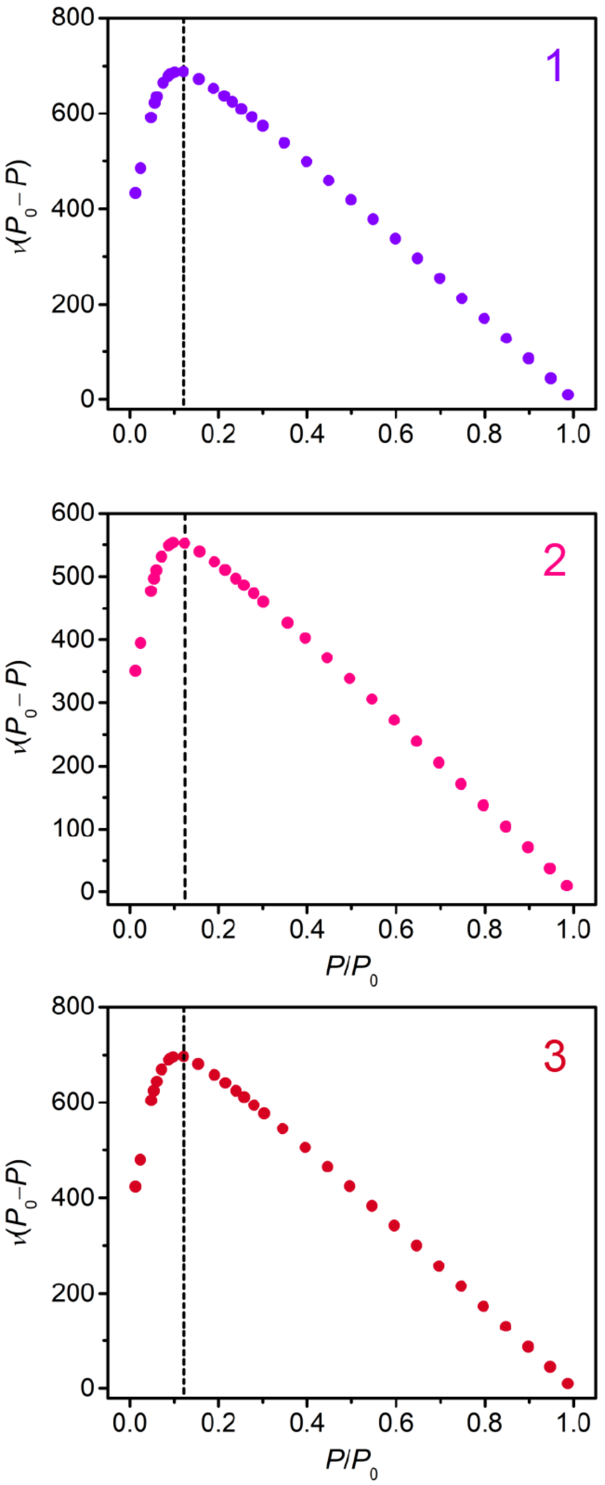

Figure S18 | Plots of $v(P_0–P)$ vs P/P_0 to determine the maximum value of P/P_0 used for fitting the BET isotherm of **1–3**, according to the first BET consistency criterion.[30] Dotted lines represent the maximum P/P_0 used for fitting.

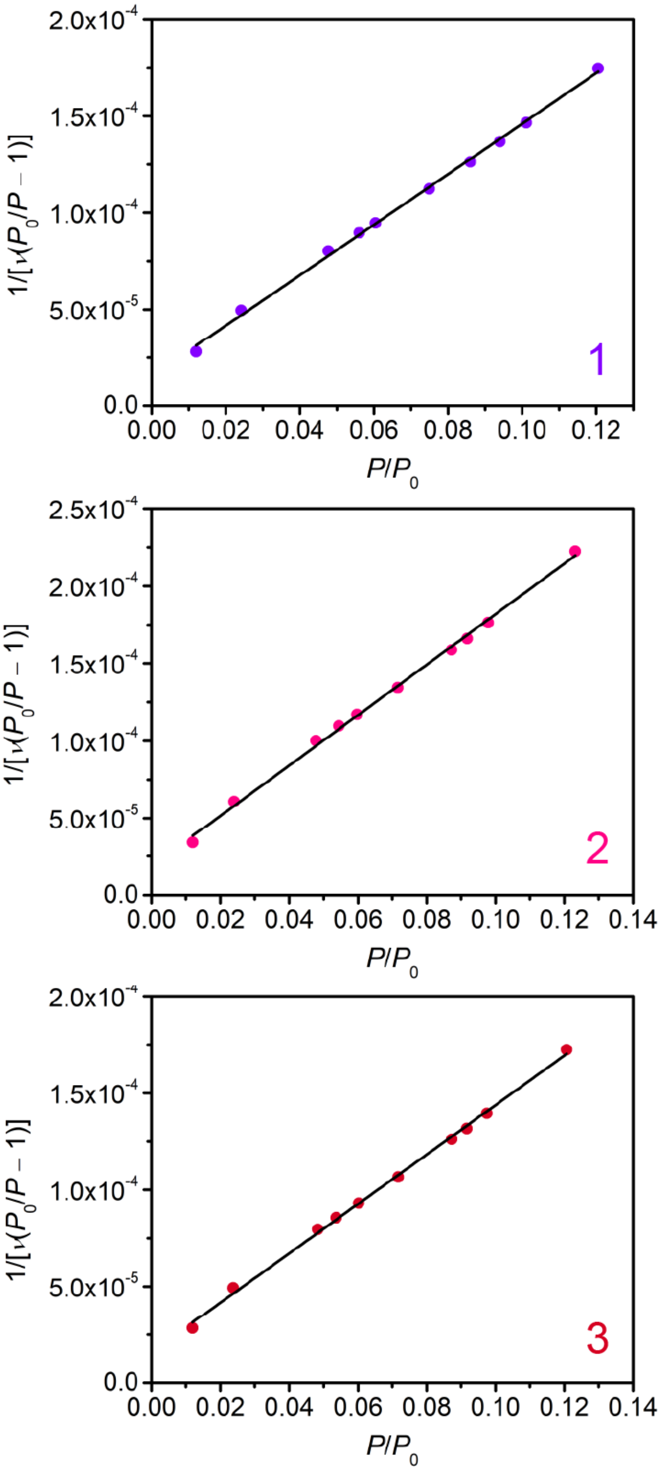

Figure S19 | Plots of $1/[v(P_0/P–1)]$ vs P/P_0 to determine the BET surface area of **1–3**. Best fit lines are shown in black, and the calculated fit parameters are reported in Table S18. The y-intercept calculated from the best fit lines fulfill the second BET consistency criterion.[30]

References

(1) D. Feng, Z.-Y. Gu, J.-R. Li, H.-L. Jiang, Z. Wei and H.-C. Zhou, *Angew. Chem. Int. Ed.,* 2012, **124**, 10453–10456.

(2) L. Li, S. Shen, R. Lin, Y. Bai and H. Liu, *Chem. Commun.,* 2017, **53**, 9986–9989.

(3) A. Yetisen, M. Qasim, S. Nosheen, T. Wilkinson and C. Lowe, *J. Mater. Chem. C,* 2014, **2**, 3569–3576.

(4) D. Feng, W.-C. Chung, Z. Wei, Z.-Y. Gu, H.-L. Jiang, Y.-P. Chen, D. J. Darensbourg and H.-C. Zhou, *J. Am. Chem. Soc.,* 2013, **135**, 17105–17110.

(5) S. Stoll and A. Schweiger, *J. Magn. Reson.,* 2006, **178**, 42–55.

(6) A. Schweiger and G. Jeschke, *Principles of Pulse Electron Paramagnetic Resonance*, Oxford University Press Inc., New York, 2001.

(7) K. L. Cunningham, K. M. McNett, R. A. Pierce, K. A. Davis, H. H. Harris, D. M. Falck, and D. R. McMillin, *Inorg. Chem.,* 1997, **36**, 608–613.

(8) P. Håkansson, T. Nguyen, P. B. Nair, R. Edge and E. Stulz, *Phys. Chem. Chem. Phys.,* 2013, **15**, 10930–10941.

(9) J. Shao, E. Steene, B. M. Hoffman and A. Ghosh, *Eur. J. Inorg. Chem.,* 2005, **2005**, 1609–1615.

(10) S. Greiner, D. Rowlands and R. Kreilick, *J. Phys. Chem.,* 1992, **96**, 9132–9139.

(11) G. Hurst, T. Henderson and R. Kreilick, *J. Am. Chem. Soc.,* 1985, **107**, 7294–7299.

(12) T. Henderson, G. Hurst and R. Kreilick, *J. Am. Chem. Soc.,* 1985, **107**, 7299–7303.

(13) J.-L. Du, K. M. More, S. S. Eaton and G. R. Eaton, *Isr. J. Chem.,* 1992, **32**, 351–355.

(14) J.-L. Du, G. R. Eaton and S. S. Eaton, *J. Magn. Reson. Ser. A,* 1996, **119**, 240–246.

(15) A. Urtizberea, E. Natividad, P. J. Alonso, M. A. Andrés, I. Gascón, M. Goldmann and O. Roubeau, *Adv. Funct. Mater.,* 2018, 1801695.

(16) H. Chen, A. G. Maryasov, O. Y. Rogozhnikova, D. V. Trukhin, V. M. Tormyshev and M. K. Bowman, *Phys. Chem. Chem. Phys.,* 2016, **18**, 24954–24965.

(17) K. Salikhov, S.-A. Dzuba and A. M. Raitsimring, *J. Magn. Reson.,* 1981, **42**, 255–276.

(18) A. Sukhanov, M. Mamedov, K. Möbius, A. Y. Semenov and K. Salikhov, *Appl. Magn. Reson.,* 2018, **49**, 1011–1025.

(19) G. Jeschke and Y. Polyhach, *Phys. Chem. Chem. Phys.,* 2007, **9**, 1895–1910.

(20) P. P. Borbat, J. H. Davis, S. E. Butcher and J. H. Freed, *J. Am. Chem. Soc.,* 2004, **126**, 7746–7747.

(21) A. Blank, *Phys. Chem. Chem. Phys.,* 2017, **19**, 5222–5229.

(22) A. J. Fielding, S. Fox, G. L. Millhauser, M. Chattopadhyay, P. M. Kroneck, G. Fritz, G. R. Eaton and S. S. Eaton, *J. Magn. Reson.,* 2006, **179**, 92–104.

(23) G. A. Bain and J. F. Berry, *J. Chem. Educ.,* 2008, **85**, 532.

(24) D. Gatteschi, R. Sessoli and J. Villain, *Molecular Nanomagnets*, Oxford University Press Inc., New York, 2006.

(25) M. Atzori, S. Benci, E. Morra, L. Tesi, M. Chiesa, R. Torre, L. Sorace and R. Sessoli, *Inorg. Chem.,* 2017, **57**, 731–740.

(26) M. Atzori, L. Tesi, S. Benci, A. Lunghi, R. Righini, A. Taschin, R. Torre, L. Sorace and R. Sessoli, *J. Am. Chem. Soc.,* 2017, **139**, 4338–4341.

(27) A. De Vroomen, E. Lijphart, D. Prins, J. Marks and N. Poulis, *Physica,* 1972, **61**, 241–249.

(28) J. Giordmaine and F. Nash, *Phys. Rev.,* 1965, **138**, A1510.

(29) L. Tesi, A. Lunghi, M. Atzori, E. Lucaccini, L. Sorace, F. Totti and R. Sessoli, *Dalton Trans.,* 2016, **45**, 16635–16643.

(30) K. S. Walton and R. Q. Snurr, *J. Am. Chem. Soc.,* 2007, **129**, 8552–8556.

(31) *APEX2*, v. 2009; Bruker Analytical X-Ray Systems, Inc: Madison, WI, 2009.

(32) G. M. Sheldrick, *SADABS*, Version 2.03; Bruker Analytical X-Ray Systems, Inc.: Madison, WI, 2000.

(33) G. M. Sheldrick, *SHELXTL*, Version 6.12; Bruker Analytical X-ray Systems, Inc.: Madison, WI, 2000.
(34) O. V. Dolomanov, L. J. Bourhis, R. J. Gildea, J. A. Howard and H. Puschmann, *J. Appl. Crystallogr.,* 2009, **42**, 339–341.
(35) W. Morris, B. Volosskiy, S. Demir, F. Gándara, P. L. McGrier, H. Furukawa, D. Cascio, J. F. Stoddart and O. M. Yaghi, *Inorg. Chem.*, 2012, **51**, 6443–6445.
(36) J. Ren, M. Ledwaba, N. M. Musyoka, H. W. Langmi, M. Mathe, S. Liao and W. Pang, *Coord. Chem. Rev.,* 2017, **349**, 169–197.
(37) S. Øien, D. Wragg, H. Reinsch, S. Svelle, S. Bordiga, C. Lamberti, and K. P. Lillerud, *Crys. Growth Des.,* 2014, **14**, 5370–5372.
